# Electrodes with Electrodeposited Water-excluding Polymer Coating Enable High-Voltage Aqueous Supercapacitors

**DOI:** 10.34133/2020/4178179

**Published:** 2020-10-09

**Authors:** Wujie Dong, Tianquan Lin, Jian Huang, Yuan Wang, Zhichao Zhang, Xin Wang, Xiaotao Yuan, Jie Lin, I-Wei Chen, Fuqiang Huang

**Affiliations:** ^1^State Key Laboratory of High Performance Ceramics and Superfine Microstructure, Shanghai Institute of Ceramics, Chinese Academy of Sciences, Shanghai 200050, China; ^2^State Key Laboratory of Rare Earth Materials Chemistry and Applications, College of Chemistry and Molecular Engineering, Peking University, Beijing 100871, China; ^3^Department of Materials Science and Engineering, University of Pennsylvania, Philadelphia, PA 19104, USA

## Abstract

Aqueous supercapacitors are powerful energy sources, but they are limited by energy density that is much lower than lithium-ion batteries. Since raising the voltage beyond the thermodynamic potential for water splitting (1.23 V) can boost the energy density, there has been much effort on water-stabilizing salvation additives such as Li_2_SO_4_ that can provide an aqueous electrolyte capable of withstanding ~1.8 V. Guided by the first-principles calculations that reveal water can promote hydrogen and oxygen evolution reactions, here, we pursue a new strategy of covering the electrode with a dense electroplated polymerized polyacrylic acid, which is an electron insulator but a proton conductor and proton reservoir. The combined effect of salvation and coating expands the electrochemical window throughout pH 3 to pH 10 to 2.4 V for both fast and slow proton-mediated redox reactions. This allows activated carbon to quadruple the energy density, a kilogram of nitrogen-doped graphene to provide 127 Watt-hour, and both to have improved endurance because of suppression of water-mediated corrosion. Therefore, aqueous supercapacitors can now achieve energy densities quite comparable to that of a lithium-ion battery, but at 100 times the charging/discharging speed and cycle durability.

## 1. Introduction

The energy of an electrochemical cell of a linear capacitance *C* operating at a voltage *V* is ½*CV*^2^. Therefore, a modest increase in operating voltage will result in a major boost in energy. Thermodynamically, the voltage is limited by the stability of the electrolyte, and aqueous electrolytes decompose into hydrogen and oxygen at 1.23 V. Many organic electrolytes can withstand a higher voltage, but they also charge/discharge relatively slowly and are burdened with certain safety and environmental concerns [1]. So there is much interest in stabilizing H_2_O molecules, e.g., by strong solvation of cations or anions. Indeed, with Li_2_SO_4_ addition to the aqueous electrolyte, the operating voltage is raised to 1.6 V in acidic electrolyte [2] or 1.8-1.9 V in neutral one [3]. Further suppressing decomposition kinetics using a high rate [4] (e.g., reaching 2.2 V at 10 mV s^−1^, Fig. S1A-I) was also claimed, but this is impractical because during applications, hydrogen will inevitably form whenever the rate slows. Independently, the disadvantage of a small electrochemical window (ECW) is also partially mitigated by advanced carbon electrodes, such as N-doped few-layer graphene that reaches a specific capacity of 855 F g^−1^, or three times the value of activated carbon in commercial supercapacitors [2]. The aim of this work is to demonstrate a water-excluding polymer-coated advanced carbon electrode that can reproducibly operate (over 10^5^ cycles) at 2.4 V in a Li_2_SO_4_ aqueous electrolyte at both high and low rates over a pH window from 3 to 10. The resulting aqueous (symmetric) supercapacitor is capable of an energy density quite comparable to that of a lithium-ion battery, but at 100 times the charging/discharging speed and cycle durability.

**Figure 1:**
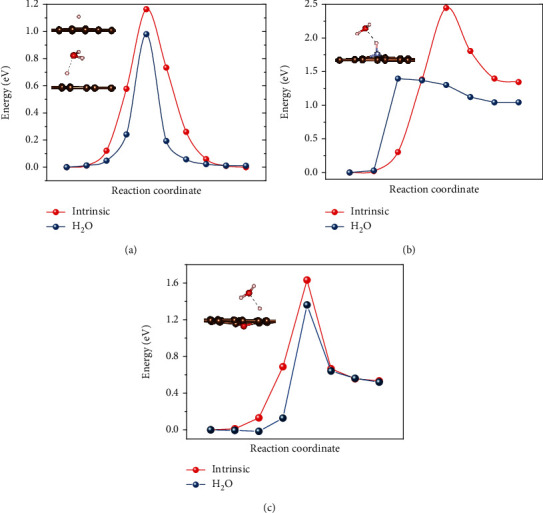
Calculated energy barrier for H diffusion by the NEB method and corresponding transition state mediated by water: (a) pristine, (b) N-doped, and (c) O-doped (with one vacancy) graphene. C, N, O, and H are represented in brown, grey, red, and white, respectively.

The main purpose of electrode coating is to suppress hydrogen evolution reaction (HER). This is because it is HER at the negative electrode that limits the ECW in a symmetric supercapacitor. (Oxygen evolution reaction (OER) at the positive electrode requires a potential *E*_OER_ (V) = 1.23–0.059 pH as opposed to *E*_HER_ (V) = −0.059 pH, thus ∣*E*_HER_ | <∣*E*_OER_∣ for pH < 10.42.) In practice, significant H_2_ gas always evolves at a cell voltage > 1.6 V whereas no O_2_ gas evolves up to 2.0 V [5], where the outward shift in potentials is due to (a) hydrogen electrosorption that locally depletes protons and increases the pH for HER and (b) a large overpotential for OER [3, 5]. Theoretically, having H_2_O in direct contact with the electrode is crucial for forming *M*H_ads_ in the so-called Volmer reaction to lower the reaction barrier at a catalytic site *M*, which allows HER to proceed at *E*_HER_. (See Materials and Methods for a summary of this and other reactions.) Strong experimental evidence also exists for H_2_O's role in more general cases: even a small amount of H_2_O vapor in ultrahigh vacuum [6–8] or H_2_O liquid in nonaqueous solvents [9–12] can enhance HER, Faradaic reactions, proton/hydrogen transfer/hopping, and water dissociation on metal and oxide electrodes—observations further supported by first-principles calculations [7, 8, 13]. We have performed first-principles calculations to obtain diffusion barriers on graphene surfaces shown in Figure 1 (also see Fig. S2-3) to confirm that indeed H^+^ hopping is easier in the presence of a H_2_O molecule: it holds on undoped, N-doped, and O-doped graphene surfaces. This has motivated us to specifically hypothesize that water is needed for HER in a broad pH range. A similar calculation of OER is also performed, which shows the consistent results (see supporting information).

Actually, the hypothesis has broader implications because the same thinking also suggests that perhaps water is needed for carbon electrode to corrode/oxidize, which has a very low thermodynamic potential (0.207 V) and is a well-known source of (a) electrode degradation during cyclic/sustained loading and (b) redox pseudocapacitance (see supporting information) [3, 5, 14, 15]. So, a coating that denies water access to electrodes could suppress both HER and corrosion. Empirically, our hypothesis is also consistent with the knowledge that naturally formed water-isolating electrode coatings are associated with an enlarged ECW. For example, in Ni-metal hydride batteries, the Ni(OH)_2_ layer allows the cell to operate at 1.34 V—without oxygen evolution until 1.44 V [16], and in lead-acid batteries, the PbSO_4_ layer enables 2 V operation—without hydrogen or oxygen evolution until 2.4 V [17]. Therefore, if a water-excluding but redox-permitting coating that is generally applicable to aqueous supercapacitors can be rationalized designed without relying on naturally formed coatings that are specific to the electrode/electrolyte combinations in question and mostly serendipitous in occurrence, the coating will not only expand the ECW and enhance charge storage but also improve the durability of carbon electrodes.

To implement this strategy, we selected a polymer coating based on the following considerations.

1. The coating should be electron-insulating but proton-conducting to allow redox reactions but not water electrolysis. Polymer coatings, already on corrosion-protected metals to suppress electron transport and in enzymatic biosensors to allow proton transport, can meet this requirement
2. To sustain high-voltage, high-rate, and high-cycle electrochemical operation, the coating should be thin yet strong enough to resist dielectric, mechanical, and chemical failure. Compared to crystalline or polycrystalline inorganics, amorphous organic polymers are dielectrically, mechanically, and chemically more robust. Moreover, as voltage increases, a relatively compliant soft-material capacitative layer will contract in thickness and expand laterally to a greater extent, thus shutting close pinholes and gaps, becoming self-healing (see supporting information)
3. Polymer coatings can be provided to any electrode-electrolyte combination thus offering a generic solution

Our selection of the specific polymer and process was further guided by the following.

- Promoting proton transport needs a sufficiently hydrophilic and anionic polymer. (Nafion, the common choice for proton exchange membrane (PEM) fuel cells, is anionic.) Moreover, the polymer coating should be dense and solid-like to allow diffusion of H^+^ but not bulkier H_2_O, H_3_O^+^, and Li^+^-solvated H_2_O
- Covering very large specific areas on advanced electrodes needs a conformal coating polymer. Because the electric field is naturally concentrated at geometric asperities (the lightening-rod effect), electrically triggered *in situ* polymerization of monomers can best form a uniform, pinhole-free electrode coating [18]
- A self-buffered polymer coating with an intermediate p*K*_a_ will allow full advantage of proton-mediated redox reactions to be taken while avoiding extreme pH of the expanded ECW where water stability is relatively poor

## 2. Results

**Figure 2:**
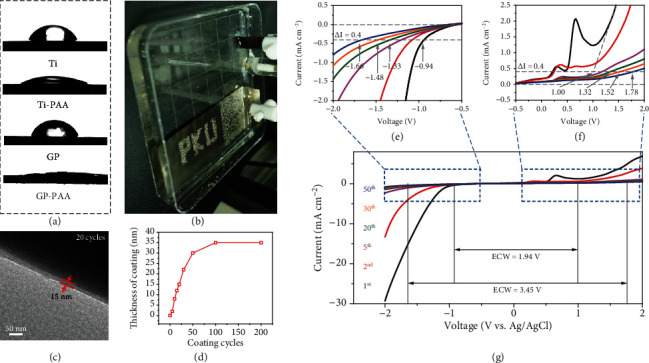
Expanding electrochemical window (ECW) by PAA coating. (a) Water droplet on uncoated/coated Ti foil and graphite paper (GP). (b) Coated electrode held at −1.8 V for 50 seconds reveals uncoated portion (PKU) highlighted by fogging from hydrogen bubbling. (c) Corresponding (120 keV) transmission electron microscope (TEM) images of the PAA layer on CCF after 20 cycles. (d) PAA layer thickness versus number of cycles. Cycling CCF electrode in 2 M Mg:2AA solution by cyclic voltammetry (CV) cycles between −2.0 and 2.0 V at the scan rate of 50 mV s^−1^ expanded ECW, evident from the enlarged view of (e) cathodic and (f) anodic extremes of (g) electrochemical stability scan. (d–f) Used three-electrode configuration with reticulated vitreous carbon counter electrode and Ag/AgCl reference electrode.

After the preliminary screening (Fig. S4), we focused on polyacrylic acid (PAA, (C_3_H_4_O_2_)_*n*_), a weak acid with p*K*_a_ = 4.7 [19]. PAA is compatible with electrochemical operations and is already used as an electrode binder [20–23] and a solid/quasisolid electrolyte [21, 24–30]. It is also an electron insulator but a proton conductor, with a proton conductivity of ~1.1 × 10^−6^ S cm^−1^ at 25°C (Fig. S5) that is fast enough for short-range proton transport in thin coatings, ca. 10 nm. We performed electrodeposition in a three-electrode cell containing an aqueous solution of Mg acrylate salt (2 M) that provides acrylic acid (AA, CH_2_ = CHCOOH) monomer, along with a reticulated vitreous carbon counter electrode and an Ag/AgCl reference electrode. To receive a coating, three inert working electrodes were initially used: conductive carbon felt (CCF), polished Ti foil (stainless steel and gold also used but for brevity, their data were not included here), and graphite paper (see Materials and Methods). The coating readily rendered the electrode surface hydrophilic (Figure 2(a)), it suppressed H_2_ bubbling at −1.8 V that otherwise would have caused fogging on an uncoated electrode (Figure 2(b)). TEM images of freshly coated and 2000 cycled CCF electrodes show that the PAA coating layers are ~15 nm in both, indicating it was apparently robust during cycling in 2 M Li_2_SO_4_ electrolyte (Fig. S6).

To obtain coating (Figures 2(c) and S7), the working electrode was cycled between −2.0 and 2.0 V at the rate of 50 mV s^−1^. The thickness of the PAA coating layer can be controlled spanning over 2 nm to 35 nm along with the CV cycles from 5 to 200 cycles (Figures 2(d) and S7). This self-limiting feature in electrocoating is common to an electron-insulator coating, because the tunneling current exponentially decreases with thickness [18]. This is seen in Figures 2(e)–2(g) (also see Fig. S8A-B for deposition on Ti and graphite paper), where the thickness-dependent ECW is defined as the voltage at a threshold current density of Δ*I* = 0.4 mA per square centimeter [31]. (This was calculated based on the macroscopic size of the electrode. For CCF, which has an electrode area of ~545 cm^2^ per cm^2^ of the macroscopic size, as measured in Fig. S9A, the actual current density is 545x lower.) Nitrogen adsorption-desorption isotherms of uncoated and PAA coated (50 cycles) CCF electrodes were also performed, as shown in Fig. S9A-B. The specific surface area of uncoated and PAA-coated (50 cycles) CCF electrodes is 4.46 m^2^ g^−1^ and 2.99 m^2^ g^−1^, respectively. This suggests that the PAA coating layer may reduce the specific surface area of carbon materials because the PAA layer will fill partial pores of carbon materials that N_2_ cannot be absorbed. Pore size distribution (Fig. S9C-D) shows that after coating PAA, the ratio of the micropores of the CCF electrode is reduced, meaning that PAA may get into the micropores. Considering that the coated PAA itself contributes to some surface area yet the entire electrode's specific surface area is reduced, the PAA layer is dense but not porous. We also repeated the electrodeposite procedure using PAA instead of AA, but it did not make the electrode hydrophilic nor did it enlarge ECW (Fig. S10). Therefore, PAA cannot coat the electrode and it is essential to have AA monomers in electrodeposition to utilize electropolymerization that converts AA in the electrolyte to the PAA coating on the electrode. Indeed, researchers on PAA electrolyte and binder have never reported finding PAA coating or ECW enlargement [20–23, 30].

**Figure 3:**
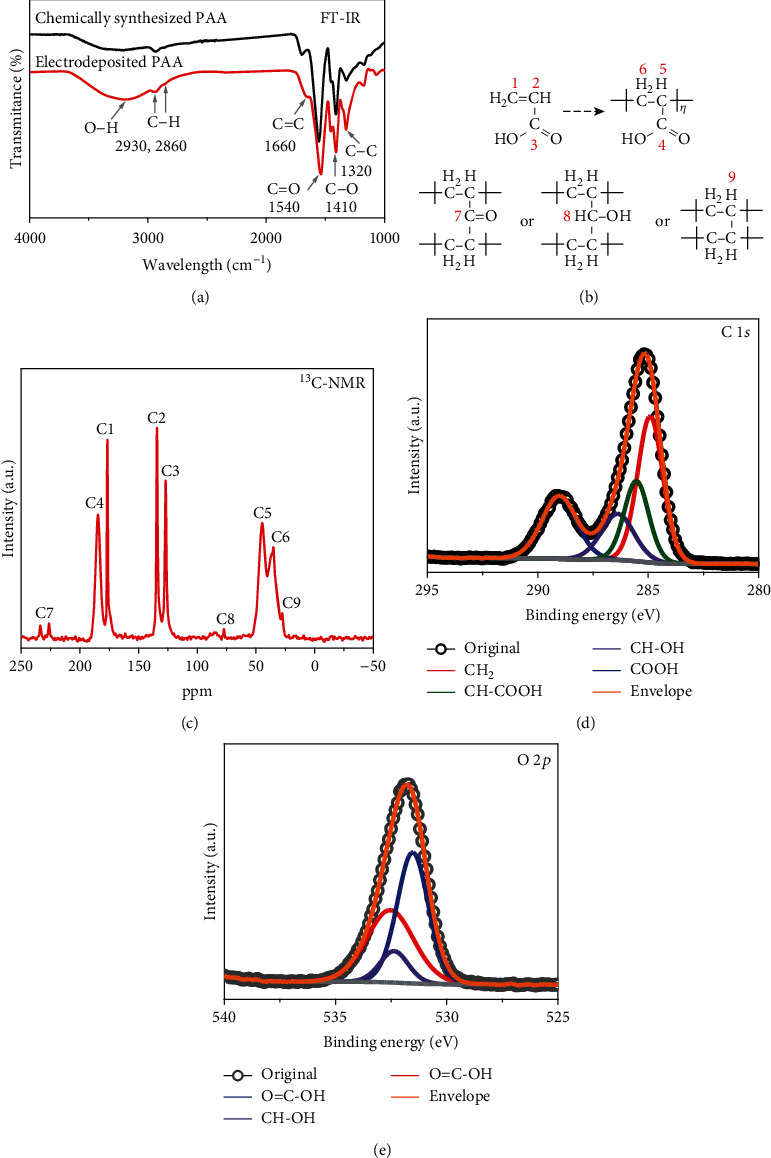
Structure of PAA coating. (a) FT-IR reflection spectroscopy of chemically synthesized (black) and electrodeposited PAA (red). (b) Labeled chemical structures of AA, PAA, and three crosslinking possibilities in the lower panel. (c) Solid-state ^13^C-NMR spectra of PAA coating, with Mg:2AA salt added as a reference; marked C1-9 in one-to-one correspondence with labeled carbon in (b). (d) C1*s* and (e) O2*p* XPS spectra of PAA coating, showing overall fitting and deconvoluted components.

It is well known that the electrodeposition of vinyl polymer is initiated at the cathode, which provides electrons and activates free radicals to initiate polymerization [32–36]. As the electron-deficient C=C in the monomer accepts a cathodic electron, it forms a new free radical to propagate polymerization further. The coating has an FT-IR (Figure 3(a)) similar to that of a reference PAA, which we separately synthesized by a standard free-radical polymerization procedure. They both reveal a small C=C content from an unreacted monomer. Although free-radical polymerization usually yields a linear polymer, apparently some chain-chain crosslinking (see schemes in Figure 3(b)) also exists in electrodeposited PAA, as evidenced by the C7, C8, and C9 peaks in ^13^C-NMR (Figure 3(c)) and the CH-OH component in C1*s* and O1*s* X-ray photoelectron spectroscopy (XPS, Figures 3(d) and 3(e)). A small extent of crosslinking is consistent with the better coating adhesion to C-containing electrodes (CCF, graphite paper, as well as activated carbon YP-50 and N-doped graphene, etc., to be described later) than to metal electrodes (Ti and stainless steel foils, Au film), because polymer can covalently bond to C on the C-containing electrodes. Indeed, a C precoat of metal electrodes using a plasma treatment greatly improved their adhesion with subsequent PAA coating (Fig. S11A-B). Lastly, alternating the voltage polarity during deposition produced a more adherent coating (Fig. S11C). This is because while hydrogen bubbles may form in cathodic charging (Figure 2(a)) to degrade the interface, their nucleation takes time, so reversing the voltage polarity frequently will deprive incubation time, hence preventing bubble nucleation. This phenomenon was already seen in solid oxide electrochemical cells albeit at a temperature of 750 K higher [37].

Being an anionic polymer, PAA can readily incorporate cations, such as Mg^2+^ in the starting solution. Here, we use Mg other than Li because Li cannot be detected by energy dispersive spectroscopy (EDS). As shown in Fig. S12A, elemental mapping confirms a relatively uniform Mg distribution, along with C and O, on as-coated CCF. However, if such CCF is next cycled 50 times (at 50 mV s^−1^) between −1.0 V and 1.0 V in a Mg-free 0.5 M H_2_SO_4_ electrolyte, then 90% of the Mg content is gone (Figure S12B), presumably replaced by H^+^. Subsequently, Mg^2+^ can be reintroduced to the coating by repeating cyclic voltammetry (CV) for another 50 cycles, this time in 2 M MgSO_4_ electrolyte. By now, the Mg content in the coating has increased by a factor of 3 (Fig. S12C), but it is still 75% below the original value (Fig. S12A). Therefore, the majority of metal ions in the as-deposited coating is probably trapped though not structurally bound to the polymer, so they can leave and reenter coating as dictated by the electrolyte composition and electrode's voltage/polarity. This ability to accommodate cations to various extents is natural for a weak acid like PAA, which can readily protonate/deprotonate to maintain charge neutrality.

As mentioned before, carbon electrodes and their surface groups (see supporting information) are prone to oxidation at low voltage, which appeared as side-reaction peaks at 0.3 V and 0.7 V in Figures 2(e)–2(g) during electrodeposition on the CCF electrode. (Understandably, these peaks were not seen on the Ti electrode). They weakened over cycles, and their existence can be manipulated by preoxidizing or prereducing CCF (see Materials and Methods and Fig. S8C-D) that tunes the population of oxygen-containing surface groups partaking in reversible proton-mediated redox reactions. The same reactions also occur to YP-50, a carbon-based active material commonly added to the supercapacitor electrodes. Taking advantage of this feature, we prepared (see Materials and Methods) pristine, unoxidized YP-50, and preoxidized YP-50—to be called M-YP-50, which has a prominent redox peak at 0.4 V (Fig. S13A-B). They afforded two model active materials: YP-50 as an electric double-layer capacitor- (EDLC-) active material and M-YP-50 as a redox-active material. Incorporated into graphite-paper-backed CCF (denoted as CCF-GP, see Materials and Methods), the two electrodes were provided a PAA coating to enable comparison of EDLC/redox activities with largely the same electrode/active-material configuration, morphology, and microstructure.

**Figure 4:**
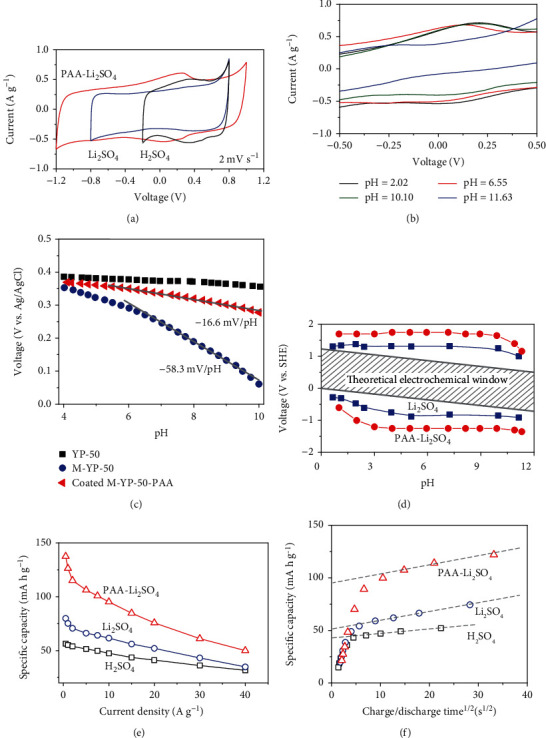
Influence of PAA coating on three-electrode electrochemical properties. Electrodes used were CCF-GP loaded by activated carbon YP-50, with and without PAA coating, some additionally modified by YP-50's preoxidation (M-YP-50). (a) CV curves scanned at 2 mV s^−1^, for YP-50-CCF-GP electrode in 0.5 M H_2_SO_4_ (label: H_2_SO_4_) and 2 M Li_2_SO_4_ (label: Li_2_SO_4_, pH~6), and PAA-coated YP-50-CCF-GP electrode in 2 M Li_2_SO_4_ (label: PAA-Li_2_SO_4_, pH~6). (b) Same as (a) for PAA-coated YP-50-CCF-GP electrode in 2 M Li_2_SO_4_ adjusted to several pH. (c) Tafel plots of electrode potential against pH at steady-state current density of 10 *μ*A cm^−2^, for YP-50-CCF-GP, and PAA-coated/uncoated M-YP-50-CCF-GP electrodes. (d) Potential for hydrogen evolution reaction and oxygen evolution reaction of uncoated (blue) and coated (red) CCF-GP electrodes in 2 M Li_2_SO_4_ electrolyte, adjusted to various pH. Shadowed band is a theoretical electrochemical window of water. Same electrode/electrolyte combinations as in (a) for (e) rate performance of specific capacity according to the galvanostatic charging/discharging test from 0.5 to 40 A g^−1^, and (f) specific capacity versus square root of half-cycle time according to CV test data from 2 to 500 mV s^−1^. Extrapolated intercept capacity is rate-independent capacity, the remainder diffusion-controlled capacity.

With an uncoated YP-50 electrode, the first-cycle CV scan in 0.5 M H_2_SO_4_ electrolyte (pH ~0) in Figure 4(a) has a small peak at 0.4 V. Consistent with its redox nature (originally from some oxygen-containing functional groups), this peak disappeared when the electrolyte was changed to 2 M Li_2_SO_4_ (pH ~6), which availed fewer protons to reactions. However, when the 2 M Li_2_SO_4_ test was repeated using a coated electrode, the peak remarkably reemerged (Figure 4(a)) as if coating can provide protons to enable redox reactions at neutral pH, which we interpret as very strong evidence of proton buffering in our PAA coating. Meanwhile, there is a progressive expansion of the ECW in Figure 4(a), first by Li_2_SO_4_ stabilization of the electrolyte, next by PAA coating, while a similar current level is maintained. Therefore, *C* is the same but ½*CV*^2^ is much higher, as intended by our design. Similar coating-resulted ECW expansion was confirmed for inert electrodes without the active material: graphite paper electrode in Fig. S14 and CCF electrode in Fig. S15.

The PAA coating enables redox reactions at higher pH: in Figure 4(b) in 2 M Li_2_SO_4_ testing under several pH, the CV curves of coated YP-50 are insensitive to the pH value, from 2 to 10, with the reactions suppressed only at pH ~12. The suppression was due to the exhaustion of protons in the PAA reservoir instead of structural damage to PAA, for the coated electrode after pH ~12 testing still reproduced the same CV curves in Figure 4(b) when tested again at lower pH (data not shown). Consistent with this result, the Tafel slope for the coated M-YP-50 electrode in Figure 4(c) over pH 4-10 is relatively flat, which is like the Tafel plot of a redox-inactive EDLC electrode—an uncoated YP-50 electrode. In fact, for an uncoated M-YP-50 electrode that is redox active but lacks proton buffering, the Tafel slope is ~58 mV/pH, exactly what one would expect for a one-electron redox reaction, 59 mV/pH. These results are supportive of our design: a self-buffered PAA coating acts like a proton reservoir, so its electrode redox reactions can proceed regardless of the pH of the surrounding electrolyte. Importantly, this self-buffering ability does not affect the ECW, which remains enlarged from pH 3 to pH 10 (Figure 4(d)). Also importantly, the self-buffered PAA coating makes it possible to support high charging/discharging rates in not only proton-rich 0.5 M H_2_SO_4_ but also proton-lean neutral 2 M Li_2_SO_4_ (Figures 4(e)–4(f)). This holds for a wide range of charging/discharging times and current densities in Figure 4(f), where the extrapolated dashed line to zero time indicates the instantaneous charge to be expected of a fully loaded PAA-coated electrode.

**Figure 5:**
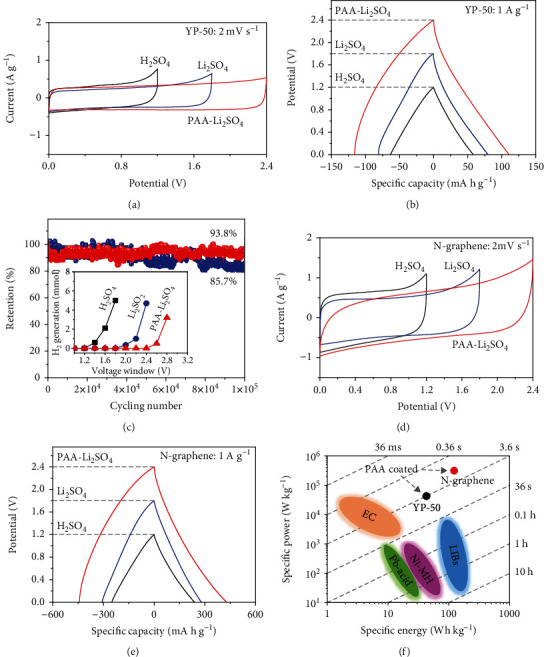
Performance of symmetric cells with PAA-coated N-doped graphene (N-graphene for short) and YP-50 electrodes. YP-50 electrode in (a–c), N-graphene-CCF-GP electrode in (d, e), with electrolyte (0.5 M H_2_SO_4_ labeled as H_2_SO_4_ and 2 M Li_2_SO_4_ at pH ~6 labeled as Li_2_SO_4_) and PAA coating indicated where applicable. (a, d) CV curves at 2 mV s^−1^. (b, e) Galvanostatic charging/discharging curves at 1 A g^−1^. (c) Retention of initial capacitance of symmetric cell with coated vs. uncoated electrodes in 2 M Li_2_SO_4_ (pH ~6). Inset: threshold voltage for water splitting determined by H_2_ accumulation (measured by gas chromatography) in a sealed symmetric cell under 24 h CV sweeping at 2 mV s^−1^. (f) Ragone plot of specific energy versus specific power for several standard devices vs. our coated YP-50 and N-graphene symmetric cells using 2 M Li_2_SO_4_ (pH ~6) electrolyte.

To assess the utility of our approach, we evaluated symmetric cells with PAA-coated electrodes with two active materials, YP-50 and N-doped graphene, whose nitrogen dopants and high conductivity are instrumental in achieving a very large redox activity [2]. Both were incorporated into CCF-GP to give an YP-50-CCF-GP electrode and an N-graphene-CCF-GP electrode (see Materials and Methods and Fig. S16), or YP-50 and N-graphene, respectively, for short. These symmetric cells with coated electrodes can withstand a voltage of 2.4 V (Figures 5(a), 5(b), 5(d), and 5(e)) when operated in 2 M Li_2_SO_4_ (pH ~6) without giving off detectable H_2_ after 24 h cycling at 2 mV s^−1^ (Figure 5(c)). They can also withstand high-voltage excursions without permanent impairment. For example, after the voltage was first ramped to 2.7 V to form H_2_, then returned to 2.4 V, an unimpaired CV curve still appeared. That is, while a higher voltage did avail tunneling electrons across the thin coating to the PAA/water interface to enable HER, it left no impression on PAA and its subsequent performance. Neither did exposure to and testing in a strong acid (pH ~2) or alkaline (pH ~11.5) damage the coating, for the ECW after such exposure/test recovered to 2.4 V when the cell was retested in a neutral Li_2_SO_4_ electrolyte (Fig. S17). This is expected because, as mentioned in Introduction, amorphous organic polymers are dielectrically, mechanically, and chemically very robust.

Overall, YP-50/N-graphene cells retained better than 94/85% of the initial capacitance after 100,000/60,000 cycles (1 A g^−1^ from 2.4 V, Figures 5(c) and S18A-B); under sustained loading of 2.4 V, their capacitance retention after 300 h was 86%/82% (Fig. S18C-D). Noting that despite a higher test voltage these are better values than those of uncoated electrodes at a lower test voltage of 1.8 V, we regard them as fully supportive of our hypothesis that a water-excluding coating can suppress degradation of carbon electrodes. Lastly, the ECW increment is again sustained over a wide range of charging/discharging conditions (Fig. S17A-B), so it is not an artifact of higher scanning rates as known to the salvation effect of Li_2_SO_4_ mentioned in Introduction. These advantages were not realized unless AA was used in electrodeposition. As in prior work [20–23, 30], directly adding PAA into the electrolyte (Fig. S10A-B) or adding PAA binder (Fig. S19) yielded no ECW-enlarging effect.

These cells achieved a very large power density in the Ragone plot in Figure 5(f). This is despite the relatively large cell resistance (8 for coated YP-50 cell and 4 for coated N-graphene cell) evident from the voltage discontinuities (Figures 5(b) and 5(e)) during polarity reversal in galvanostatic charging/discharging. Based on the weight of the active electrode material, the coated N-graphene cell having a specific capacitance of 417 mA h g^−1^ at a charge/discharge rate of 1 A g^−1^ delivers a specific power density of 237 kW kg^−1^ and a specific energy density of 127 Wh kg^−1^. Due to the high specific capacitance of our N-doped graphene and the high electrochemical window, the coated N-graphene cell shows superior than other reported carbon-based aqueous and nonaqueous supercapacitors (Table S4). For the YP-50 cell, the corresponding properties are specific capacitance of 111 mA h g^−1^, specific power density of 34 kW kg^−1^, and specific energy density of 28 Wh kg^−1^. Electrodes with a high mass loading, up to 12 mg cm^−2^, were also evaluated (Fig. S20). The specific capacity at 12 mg cm^−2^ is maintained at ~90% value of that at 1 mg cm^−2^ with coated electrodes—the uncoated ones can only maintain ~60%. Such increased mass loading allows the specific surface capacity density of the coated electrodes to reach 12 mA h cm^−2^ compared to 6 mA h cm^−2^ for uncoated electrodes, and we may again attribute the improvement to PAA serving as a proton reservoir supplying protons to the nearby active materials. The proton reservoir particularly benefits the coated N-graphene electrode allowing it to enjoy fast-rate redox reactions at neutral or close to neutral pH, thus realizing a 2.4 V ECW (Fig. S17). In contrast, without coating, the best performance of N-graphene is at pH 2 where it can take full advantage of proton-mediated redox reactions but must suffer from a considerably narrower ECW [2].

Lastly, since coating thickness is accurately and easily controlled by the number of cycles, it is an obvious parameter to optimize. Naturally, a thicker coating raises the serial resistance as evident from the potential discontinuity in galvanostatic charging/discharging curves (Figures 5(b), 5(e), S14B, and S21A-B) and the more battery-like and less capacitor-like impedance spectroscopy (Fig. S21C-E). But the increased resistance is still not enough to cause load sharing, hence shearing of the CV loops in three-electrode configurations (Fig. S22A) and symmetric cells (Fig. S22B-C) at 2 mV s^−1^. Without such complication, the CV test provides a straightforward tool to identify the optimal thickness to reap the benefit of ECW expansion without sacrificing current, capacity, and rate capability, which happens when thickness exceeds 15 nm (Fig. S21E, S22B-C, S23). But since the same high capacitance is still maintained at low current densities (e.g., 0.1 A g^−1^), the capacitance loss at high rates (Fig. S23) is not intrinsic and must be kinetic in origin, caused by sluggish diffusion/redox reaction.

## 3. Conclusion

In summary, a generic electrodeposited, electropolymerized, and electrocrosslinked polymer coating (thickness controllable PAA layer ~2-35 nm) allowing diffusion of H^+^ but not bulkier H_2_O, H_3_O^+^, and solvated H_2_O is likely to benefit many electrodes and aid their electrochemical performance. This includes enlargement of electrochemical window in voltage (2.4 V) and pH (pH 3-10), improvement of electrode stability and cyclic durability, enhanced rate performance, and multifold increases in the energy density of 127 Wh kg^−1^ and power density of 237 kW kg^−1^ based on the active materials of N-doped graphene. Exploring such an approach will help guide design and deliver new outstanding coated electrodes for fast-rate, high-capacitance energy storage.

## 4. Materials and Methods

### 4.1. Electrode Materials

This work used several basic electrodes described below. By adding active materials described in the next section to them, additional electrodes were also obtained. Conductive carbon felt (CCF) electrodes were cut from CCF (Jinlin Shuangpeng Carbon Material Technology Co., Ltd. specific resistance < 2 *Ω* · cm, fiber diameter ~10 *μ*m) into the size of 10 mm × 30 mm × ~1 mm, then washed by DI water and alcohol three times each, with 1 h ultrasonic treatment every time. To investigate the side reactions at 0.3 V and 0.7 V (Figure 2(f)), oxygen-plasma oxidation and H_2_/Ar reduction of CCF electrodes were performed as follows. To preoxidize, an electrode was placed in an oxygen-plasma reactor (Harrick Plasma Cleaner, PDC-32G-2, Ithaca, NY) in air at a radiofrequency power of 10.5 W for 10 min. To prereduce, an electrode was annealed at 500°C for 4 h in flowing H_2_/Ar (H_2_ ~10%, at 300 mL min^−1^). Ti foil electrodes were cut from a Ti foil (Shenzhen Kejin Star Technology Co., Ltd.) into the size of 10 mm × 30 mm × 0.1 mm, then polished and wiped clean before use. Graphite paper (GP) electrodes were cut from GP (Toyo Tanso Co., Ltd.) into the size of 20 mm × 40 mm × 0.4 mm, then wiped clean before use. Graphite-paper-backed CCF (CCF-GP) electrodes were prepared by bonding CCF disks (*Φ* = 14 mm, thickness ~1 mm) to graphite paper (GP, 20 mm × 40 mm × 0.4 mm) using graphite-conducting resin (YH-A018, YiHui Adhesive Co., Ltd., Dong Guan), as shown in Fig. S16. In three-electrode cells, reticulated vitreous carbon (RVC) was used as the counter electrode and Ag/AgCl electrode as the reference electrode.

### 4.2. Active Materials

Activated carbon YP-50 (Kuraray Chemical) was used without further processing. To fabricate electrodes, YP-50 powders were first dispersed in an ethanol solution of 0.5% Nafion (binder) to form a slurry with a solid loading of 20 mg mL^−1^; then, the slurry was added dropwise to a CCF-GP electrode. The structure was dried under vacuum at 80°C for 2 h to remove ethanol to obtain an YP-50-CCF-GP electrode. Nitrogen-doped graphene (N-graphene for short) came from a previous study in which it was identified as OMFLC-N, S1 [2]. The N-graphene loaded electrodes (N-graphene-CCF-GP) were fabricated in the same manner as YP-50-CCF-GP electrodes. Modified YP-50-CCF-GP (M-YP-50-CCF-GP for short) electrodes are preoxidized electrodes prepared in the following way. A YP-50-CCF-GP electrode was subject to 200 cycles of cyclic voltammetry (CV) scanning in 1 M H_2_SO_4_ aqueous electrolyte at 50 mV s^−1^ (0 V–1.2 V). This caused oxidation of YP-50 and pseudocapacity with a prominent redox peak at 0.4 V, as shown in Fig. S13.

### 4.3. Three-Electrode Cell

In a typical setting of a three-electrode cell, the working electrode was held by a high-conductivity Pt clamp, along with a RVC counter electrode and an Ag/AgCl reference electrode. For electrolyte, either 0.5 M H_2_SO_4_ (pH 0) or 2 M Li_2_SO_4_ (pH ~2-11.5) solutions were mostly used. This setting was employed not only for electrochemical characterization but also for electrodeposition of polymer coating as described below.

### 4.4. Solutions for Electrode Coating

The Mg:2AA solution was prepared by adding 8.06 g MgO (AR Sinopharm Chemical Reagent Co., Ltd.) to 100 mL deionized water with stirring, followed by dropwise addition of 28.8 mL acrylic acid (AA, with 0.05-0.1% polymerization inhibitor, hydroquinone, Xilong Chemical Co., Ltd.). The amber solution obtained after cooling contains 2 mol L^−1^ Mg:2AA, with a pH ~5. Other Mg:2AA solutions with different concentrations were similarly obtained.

Aqueous solutions of three other polymers, polyvinylidene fluoride (PVDF, Alfa Aesar)/N-methyl pyrrolidinone (NMP, Xilong Chemical Co., Ltd.), polyvinyl alcohol (PVA-224, Aladdin, MW ~205,000), and polyethylene glycol (PEG, Aldrich, MW ~20,000), were prepared by dissolving their powders in DI water to obtain solutions containing 20 mg mL^−1^ polymer. PVDF and PEG are soluble at room temperature. The dissolution of PVA in DI water was at 90°C.

### 4.5. Electrodeposition of PAA

Thin-film coating of PAA on the surface of the electrode was obtained by electrochemical deposition that also caused *in situ* polymerization. Specifically, a three-electrode cell was assembled using 2 mol L^−1^ Mg:2AA solution as an electrolyte, the electrode to be coated as the working electrode, an Ag/AgCl reference electrode, and a RVC counter electrode. In order to balance the electrochemical performance and the thickness, we tried a series of electrochemical experiments to control the electrodeposite of PAA. For example, we tried to control voltage, current, time, and so on. Based on the preliminary experiment result, we finally chose the CV electrodeposite method, which can form a uniform PAA layer on the carbon materials' surface. Theoretically, acrylic acid can polymerize on the electrode surface at negative potential due to the electron-induced C=C polymerization (free radical polymerization). However, using constant voltage or current underpotential deposition may result in too fast electrodeposition and form a depletion layer near the electrode; thus, the deposited PAA layer is not uniform enough. Using the CV method with the voltage spanning over -1.6 V to 1.6 V and the scan rate of 50 mV s^−1^ can obtain a uniform PAA layer. It is because PAA can form on the electrode surface at a negative potential, but since the scan rate is fast, the deposited PAA layer is very thin (~0.4-0.8 nm for each cycle, as shown in Fig. S7). Besides, the depletion layer near the electrode will not form because of the positive potential range for each cycle, thus the deposition speed for each cycle is quite stable until the PAA layer become too thick. The thick PAA layer (>35 nm) can block the electron out from the electrode even at a negative potential; thus, the free radical polymerization of PAA is stopped. According to our research, the optimized PAA layer thickness is ~15 nm (20 CV cycles), which can expand the ECW to ~2.4 V and maintain the fast charge-discharge properties (Fig. S23). Other protocols were also experimented, e.g., in Fig. S11C.

### 4.6. Synthesis of PAA

Chemically synthesized PAA was obtained by a typical free radical reaction. To be specific, 1 g (NH_4_)_2_S_2_O_8_ (AR, Aladdin) and 5 g AA were dissolved into 100 mL DI water; after that, 8 g isopropanol was added into the above solution, which was heated to 65-70°C. A second solution was made of 40 g AA, 2 g (NH_4_)_2_S_2_O_8_, and 40 mL DI water, and it was dropwise added to the first solution over ~30 min. After thorough mixing, the solution was refluxed at 94°C for 1 h to obtain PAA. The mixture was dialyzed and freeze-dried.

### 4.7. Symmetric Cells

Symmetric devices have two identically configured electrodes (identical in composition, size, weight, and capacitance) as cathode and anode. For example, two identical active-material-loaded electrodes were first soaked in DI water, then coated by PAA using cyclic voltammetry protocol in 2 M Mg:2AA electrolyte from −1.6 V to 1.6 V in the three-electrode configuration. The number of CV cycles is a control parameter: for active-material-loaded CCF-GP electrode (mass loading ~1 mg cm^−2^), we found 20 cycles optimal. The coated electrodes were washed in DI water to remove unreacted monomer and electrolyte. Finally, the two electrodes were separated by a glass fiber mat (Whatman) and heat-sealed by an Al-plastic film after adding the electrolyte (2 M Li_2_SO_4_, ~0.5 mL). An assembled soft package cell is shown in Fig. S16. Symmetric devices with uncoated electrodes were similarly fabricated without the PAA-coating step.

### 4.8. Computational Method

Our first-principles calculations were motivated by the following understanding of the hydrogen evolution reaction (HER) mechanism [38, 39]. In an acidic environment with proton adsorption at a catalyst site *M* on the electrode forming a catalytic intermediate *M*H_ads_, the HER starts with

(1) $$\begin{array}{c} \text{Volmer reaction}:M+{\text{H}}_{3}{\text{O}}^{+}+{\text{e}}^{-}↔M{\text{H}}_{\text{ads}}+{\text{H}}_{2}\text{O} \end{array}$$

This is followed by H_2_ release via

(2) $$\begin{array}{c} \text{Heyrovsky reaction}:M{\text{H}}_{\text{ads}}+{\text{H}}_{3}{\text{O}}^{+}+{\text{e}}^{-}↔M+{\text{H}}_{2}+{\text{H}}_{2}\text{O} \end{array}$$

or

(3) $$\begin{array}{c} \text{Tafel reaction}:2M{\text{H}}_{\text{ads}}↔2\;M+{\text{H}}_{2} \end{array}$$

In neutral and alkaline environments, reaction (3) remains viable but reactions (1) and (2) must change to reactions (4) and (5).

(4) $$\begin{array}{c} \text{Volmer reaction}:M+{\text{H}}_{2}\text{O}+{\text{e}}^{-}↔\;M{\text{H}}_{\text{ads}}+{\text{OH}}^{-} \end{array}$$

(5) $$\begin{array}{c} \text{Heyrovsky reaction}:M{\text{H}}_{\text{ads}}+{\text{H}}_{2}\text{O}+{\text{e}}^{-}↔M+{\text{H}}_{2}+{\text{OH}}^{-} \end{array}$$

Thus, having H_2_O in direct contact with the electrode is crucial for forming *M*H_ads_, which is needed to lower the reaction barrier to allow HER to proceed at the thermodynamic potential *E*_HER_. Therefore, we have undertaken a systematic computational study on the role of H_2_O in electrode reactions. Our study also included an oxygen evolution reaction (OER) to take advantage of the understanding of such a reaction in the literature [40]. These reactions require the diffusion of H^+^ and OH^−^, respectively, across material surfaces. On the surfaces of noble metals and oxides, such diffusion is already known to be aided by the presence of a H_2_O molecule [8].

All calculations were performed at the level of density functional theory (DFT) using the Vienna Ab initio Simulation Package (VASP) [41]. A plane-wave basis and projector-augmented wave method (PAW) pseudopotentials were used [42]. The Perdew-Burke-Ernzerhof (PBE) generalized gradient approximation (GGA) was adopted to treat exchange-correlation effects [43]. A cutoff of 520 eV was imposed on the kinetic energy, and the accuracy of the total energy was set to be 10^−8^ eV. Atomic and lattice parameter relaxations were optimized by a conjugate-gradient (CG) algorithm with an imposed numerical threshold of 0.005 eV/Å. We also considered the van der Waals interaction using the DFT-D2 method of Grimme [44] in the present study. A 6 × 6 × 1 supercell of graphene (72 atoms) was built for the adsorption of H^+^ and OH^−^, where the free energy of adsorption (e.g., for hydrogen, Δ*G*_H_) is defined as Δ*G*_H_ = Δ*E*_H_ + Δ*E*_ZPE_–*T*Δ*S*_H_ [45]. Here, Δ*E*_H_ is the chemisorption energy of hydrogen atom, defined as the ground-state energy difference of the adsorbed hydrogen and a free hydrogen (on graphene, Δ*E*_H_ is positive meaning an endothermic reaction consistent with the literature [45]). In addition, Δ*E*_ZPE_ is the correction of zero-point energy, *T* is the temperature (300 K here), and Δ*S*_H_ is the entropy between the adsorbed hydrogen and free hydrogen (in the gas phase). As the vibrational entropy of H in the adsorbed state is small, the entropy of adsorption is Δ*S*_*H*_ ≈ −1/2*S*0 *H*2, where *S*0 *H*2 is the entropy of H_2_ in the gas phase at standard conditions. Moreover, since our study is focused on the effect of H_2_O on surface diffusion of H^+^/OH^−^, the same method was used to quantify the tendency of H_2_O to adsorb onto graphene surfaces in terms of the binding energy, *E*_b_, defined as *E*_b_ = *E*_g/water_–*E*_water_–*E*_g_. Here, *E*_g/water_ is the total energy of the water-graphene system after geometric relaxation, *E*_water_ is the total energy of a (stand-alone) water molecular, and *E*_g_ is the total energy of a (stand-alone) graphene, so a more negative *E*_b_ indicates a stronger tendency for H_2_O adsorption. Lastly, to study the energy barriers of H^+^ and OH^−^ migration, we employed the nudged elastic band (NEB) [46] method implemented in VASP with eight image structures between the reactant and the product.

### 4.9. Material Characterization

Standard material characterization included the following. Nitrogen adsorption-desorption isotherms at 77 K were measured by a Micromeritics Tristar 3000 system using vacuum-degassed samples (180°C for at least 6 h). These isotherms were used to calculate (a) the specific surface area by the Brunauer-Emmett-Teller (BET) method and (b) the pore volume and pore size by the Barrett−Joyner−Halenda (BJH) method. For microscopy, scanning electron microscopy (SEM) images were obtained using a field emission Magellan 400 microscope (FEI Company), and transmission electron microscopy (TEM) was conducted using a JEOL 2011 microscope. X-ray photoelectron spectroscopy (XPS) was collected in a RBD upgraded PHI-5000C ESCA system (Perkin Elmer) with Mg *Kα* radiation (*hν* = 1253.6 eV). Cyclic voltammetry (CV) tests and constant (galvanostatic) charge-discharge (CC) tests were performed using an electrochemical analyzer, CHI 660E, under ambient conditions. Electric impedance spectroscopy (EIS) was performed with an excitation amplitude of 10 mV, scanned from 10 MHz to 100 kHz. Fourier transform infrared (FT-IR) reflection spectroscopy was performed in a Spectrum Spotlight 200 FT-IR microscopy (Spotlight 200, PE, US). The solid-state ^13^C-NMR spectrum was measured at 400 MHz in a WB Solid-State NMR Spectrometer (AVANCE III, Bruker, Switzerland). Proton conductivity of a commercial additive-free PAA (Macklin, MW~ 5,000, 50% aqueous solution, dried at 70°C before the measurement) was measured in a stainless steel/PAA/stainless steel cell (having a PAA electrolyte of Φ = 15.5 mm and thickness = 1 mm between two stainless steel disk electrodes) using AC impedance spectroscopy between 0.01 Hz and 100 kHz as shown in Fig. S5. With only proton mobile, the proton conductivity *σ* can be estimated by *σ* = *h*/(*R* × *S*), where *h* is the thickness of the PAA film, *R* is the total resistance obtained from the crossover point of the semicircle and abscissa at the high-frequency end ~70,000 *Ω*, and *S* is the disk area (*S* = *πr*2, *r* is the radius of the PAA disk). The proton conductivity obtained in this way was ~1.1 × 10^−6^ S cm^−1^ at 20°C.

## Supplementary Materials

Supplementary Materials Computational results and discussion (Table S1-3). Table S4: comparison of some related carbon-based aqueous and nonaqueous supercapacitors. Self-healing, oxidation of carbon electrodes and their oxygen-containing surface groups, electrochemical data, and supplementary figure (Figure S1-S23) are available in Supplementary Materials.
