# Supplementary material for: Electrodes with Electrodeposited Water-excluding Polymer Coating Enable High-Voltage Aqueous Supercapacitors
Source: Research (Wash D C). 2020 Oct 9;2020:4178179. doi: 10.34133/2020/4178179 (PMC7568819; doi:10.34133/2020/4178179)
Supplement: Supplementary Materials — Computational results and discussion (Table S1-3). Table S4: comparison of some related carbon-based aqueous and nonaqueous supercapacitors. Self-healing, oxidation of carbon electrodes and their oxygen-containing surface groups, electrochemical data, and supplementary figure (Figure S1-S23) are available in Supplementary Materials. [file 4178179.f1.docx]

**Supporting Information**

Electrodes with Electrodeposited Water-Excluding Polymer Coating Enables High-Voltage Aqueous Supercapacitors

**Wujie Dong,^1#^ Tianquan Lin,^2#^ Jian Huang,^2^** **Yuan Wang,^2^ Zhichao Zhang,^3^ Xin Wang,^1^* Xiaotao Yuan,^1^ Jie Lin, ^2^ I-Wei Chen,^3^* and Fuqiang Huang^1,2^***

^1^ State Key Laboratory of High Performance Ceramics and Superfine Microstructure, Shanghai Institute of Ceramics, Chinese Academy of Sciences, Shanghai 200050, P. R. China

^2^ State Key Laboratory of Rare Earth Materials Chemistry and Applications, College of Chemistry and Molecular Engineering, Peking University, Beijing 100871, P. R. China

^3^Department of Materials Science and Engineering, University of Pennsylvania, Philadelphia, PA 19104, USA

^#^ WJ. Dong and TQ. Lin contributed equally to this work.

* Correspondence should be addressed to I-Wei Chen: [iweichen@seas.upenn.edu](mailto:iweichen@seas.upenn.edu) and Fuqiang Huang: [huangfq@mail.sic.ac.cn](mailto:huangfq@mail.sic.ac.cn)

**Computational Results and Discussion**

Guided by the study of Jiao et al.,[1] we used the structural models for graphene shown in **Fig. S2**. Adsorption and diffusion of H^+^ and OH^-^ was investigated on three graphene surfaces: a pristine one, and two defective ones doped by N or O. In the latter two, C site 1 refers to the nearest C of the dopant, and C site 2 is the second nearest C of the dopant. Note that in **Fig. S2** O-doping creates an additional C vacancy next to the substitutional O, whereas N-doping allows a graphitic N (denoted as NQ) to substitute for a graphitic C without creating any vacancy. While admittedly oversimplified, these models turn out to still provide some indication of the beneficial effect of H_2_O assistance on H^+^/OH^-^ diffusion.

We first describe the result of H_2_O binding energy in **Table S1**. A negative binding energy is found on all surfaces of graphene, regardless of whether doped (by N or O) or not (i.e., pristine). Moreover, a negative binding energy is found after the surfaces are pre-adsorbed by an atomic H or OH, and it actually becomes more negative on surfaces with pre-adsorbed OH. In this respect, it is interesting to note that there is a recent report that found the graphene surface to be intrinsically hydrophilic, but adsorbed hydrocarbon can make it hydrophobic.[2]

We next calculated the free energy of H^+^ adsorption to the surface, with and without the participation of an additional H_2_O molecule. The absorption free energy of H^+^ on pristine graphene and O-doped graphene increases with H_2_O participation, while the opposite is true on N-doped graphene (**Table S2**.) In contrast, absorption free energy of OH^-^ in **Table S3** always decreases with H_2_O participation. They are, however, always positive in agreement with the literature.[3] Therefore, these graphene surfaces are not too “sticky” for the absorbed H^+^ or OH^-^ to move around. Meanwhile, the positive values are not too extreme to make it entirely impossible for H^+^ and OH^-^ to absorb, which would also make surface diffusion unlikely.

**Table S1** Binding energies of H_2_O adsorbed to graphene and H/OH-pre-adsorbed graphene, the latter denoted as H*/OH*-graphene.

|  | Binding site | Energy (eV) Graphene | Energy (eV)  H*-Graphene | Energy (eV)  OH*-Graphene |
| --- | --- | --- | --- | --- |
| Pristine | all C sites | -0.298 | -0.202 | -0.340 |
| N-doped (NQ) | C site 1 | -0.160 | -0.532 | -0.297 |
|  | C site 2 | -0.160 | -0.226 | -0.377 |
| O-doped | C site 1 | -0.228 | -0.137 | -0.337 |
|  | C site 2 | -0.228 | -0.171 | -0.437 |

**Table S2** Absorption free energy of H^+^ at various binding sites on graphene.

|  | Binding site | Energy (eV)  Without H_2_O | Energy (eV)  With H_2_O |
| --- | --- | --- | --- |
| Pristine graphene | all C sites | 0.766 | 0.862 |
| N-doped graphene (NQ) | C site 1 | 0.526 | 0.484 |
|  | C site 2 | 0.958 | 0.893 |
| O-doped graphene | C site 1 | 0.485 | 0.573 |
|  | C site 2 | 1.018 | 1.073 |

**Table S3** Absorption free energy OH^-^ at various binding sites on graphene.

|  | Binding site | Energy (eV)  Without H_2_O | Energy (eV)  With H_2_O |
| --- | --- | --- | --- |
| Pristine graphene | all C sites | 1.686 | 1.644 |
| N-doped graphene (NQ) | C site 1 | 1.318 | 1.181 |
|  | C site 2 | 1.820 | 1.604 |
| O-doped graphene | C site 1 | 1.975 | 1.867 |
|  | C site 2 | 1.146 | 0.938 |

Although absorption free energy, which is a thermodynamic quantity, is often used to evaluate the HER efficiency because in noble metal systems a known linear correlation between the adsorption free energy and the kinetic behavior (e.g., reaction barrier) holds,[4] such correlation is not a priori and can fail in some cases.[5] Therefore, we used the nudged elastic band (NEB) method [6] to determine the energy barrier of H^+^ diffusion from one C site (e.g., *C1) to a neighboring one (e.g., *C2), a process represented by the following reaction:

H_ads_(*C1) + (*C2) → (*C1) + H_ads_(*C2) (S6)

As shown in **Fig. 1A,** on the H_2_O-free surface of an undoped (pristine) graphene, the lowest barrier is ~1.2 eV, which is close to the value reported for H^+^ diffusion on oxide surfaces.[7] With H_2_O participation, the barrier decreases by 15%. Such a modest barrier lowering (of about 0.2 eV according to **Fig. 1A**) is already significant for the diffusion coefficient *D*_S_ = *av**exp(−*E_a_*/*k*_B_T). Here, *v**, *E*_a_, and *a* are the attempt frequency, the activation barrier and the jump distance, respectively. This is because, at 300 K (*k*_B_*T*=0.025 eV), decreasing *E*_a_ by 0.2 eV increases *D*_S_ by a factor of exp(8) or 3000 times. In **Fig. 1A**, this enhancement is no doubt related to water assistance, for we have also verified that the transition state complex (shown in **Fig. 1A**) is essentially a H_3_O^+^. Such a complex was previously reported for H^+^-H_2_O diffusion on oxide surfaces.[8]

On N-doped graphene, the introduction of a H_2_O modestly reduces the absorption free energy of H^+^ at both C site 1 and C site 2, by about 0.05 eV, which is even less than above (see **Table S2**). Yet H_2_O has a large effect on the energy barrier of H^+^ diffusion, which is 2.5 eV without H_2_O assistance and 1.5 eV with H_2_O assistance (i.e., H_3_O diffusion) in **Fig. 1B**. Clearly, this will lead to a huge increase of the diffusion coefficient.

Interestingly, on O-doped graphene, the absorption free energy for H^+^ at different binding sites is actually increased by water participation (**Table S2**). However, as shown in **Fig. 1C**, the energy barrier of diffusion still decreases by ~0.2 eV with the introduction of a H_2_O. The transition state again involves a H_3_O^+^, which is made of a pre-adsorbed H_2_O and a nearby proton, the latter moving between two one C sites. (In our calculation, we provided an extra proton to the system.) So, in this case, the advantage of H_2_O participation is not at all foreseen by the absorption free energy: The transition state dynamics is much more important.

For OER, migration of OH^-^ across the material surface is important. The energy barriers for OH^-^ migration from NEB are summarized in **Fig. S3.** For all three surfaces, the energy barrier decreases by ~0.3 eV after allowing H_2_O participation. Remarkably, at the transition state the H_2_O molecule in the H_2_O-OH^-^ complex rotates to a larger extent than the H_2_O molecule in the H_3_O^+^ complex does in the transition state of H^+^ diffusion. This suggests it is such distortion that plays a crucial role in the transition state dynamics, and because of it the dynamics can exert an essential influence on diffusion barrier to decouple diffusion kinetics from adsorption thermodynamics. As a final note, on the surface of N-doped graphene, OH^-^ at the transition state is not at the N-site; OH^-^ actually binds to a C site neighboring N.

**Self-healing**

Self-healing happens during coating and in subsequent electrochemical testing. During coating, the rate of electrodeposition and electropolymerization is known to increase nonlinearly with the current density. If there is any pinhole or crack developed in the coating, then it will become a current concentrator, which will greatly accelerate electrodeposition and electropolymerization. Therefore, the pinhole or crack is rapidly plugged, after that the rate of electrodeposition and electropolymerization returns to normal. This well-known mechanism is thought to be responsible for the generally uniform and impervious coating created by electrodeposition and electropolymerization of insulating polymer, from monomers. In essence, the above process is a negative feedback loop that serves to regulate the coating process to make it uniform and dense.

During subsequent electrochemical testing, self-healing may be considered by asking whether a 1 nm wide crack in a 10 nm thick coating can be shut by a voltage drop of 2 V across the coating. Self-healing arises because: (1) the voltage *V* will cause the coating thickness to shrink, in the same way that the gap in a parallel-plate capacitor will shrink due to the Coulomb attraction between two oppositely charged plates, (2) the thickness contraction will cause a lateral expansion of polymer, (3) the lateral expansion will shut the crack. For an order of magnitude estimate, we will assume an incompressible coating and ignore stress variation in the coating. (If coating is compressible and the Poisson contraction is considered instead, then the lateral expansion will be less. On the other hand, if lateral expansion is held at zero at the coating/electrode interface because of strong bonding, then the lateral expansion at the other side, the free surface side of the coating, will be more. Therefore, the two effects tend to cancel each other.) For a coating of a thickness *d* that sees an electric field *E*, the force on the surface charge provides the compressive stress, *s*=ε*E*^2^, where ε is permittivity of coating, and *E=V/d*. With *Y* as Young’s modulus of the coating, this gives a compressive strain *e=s/Y*, which may be equated to the lateral expansion strain. The total lateral displacement between two cracks of a spacing *L* apart is thus *a*=*Le*=ε*LV*^2^/*Yd*^2^. Letting *L*=10 μm, *V*= 2 V, *d*=10 nm, ε=2ε_o_=2x8.854x10^-12^ Farad/m where ε_o_ is the permittivity of free space (i.e., the relative dielectric constant is 2, which is typical for a polymer), and *Y*= 10 GPa, which is on the very high end for a polymer, we obtain *a*=0.7 nm. Therefore, there is a viable mechanism for crack healing.

**Oxidation of carbon electrodes and their oxygen containing surface groups**

Electrochemical oxidation/corrosion of carbon materials in the presence of water may proceed by the following reaction:

C + 2H_2_O → CO_2_ + 4H^+^ + 4e^−^ (S7)

The standard potential of Reaction (S7) is only 0.207 V in aqueous acidic electrolyte. But the reaction above was believed to be very slow at this low potential.[9] The actual reaction maybe more complex. Jarvi *et al.[10]* stated that surface oxide generation in acidic electrolytes involves the general steps of oxidation of carbon in the lattice structure (Reaction (S8) below) followed by hydrolysis (Reaction (S9) below) and finally gasification of oxidized carbon to CO_2_ (Reaction (S10) below). They also proposed a generic stepwise mechanism of surface oxide formation and CO_2_ evolution as schematically shown in Reaction (S11) below where water is understood to be the source of oxygen. (In these reactions, the subscript s denotes surface species.)

C_s_ → C_s_^+^ + e^−^ (S8)

C_s_^+^ + ½H_2_O → C_s_O + H^+^ (S9)

2C_s_O + H_2_O → C_s_O + CO_2_ + 2H^+^ + 2e^−^ (S10)

R-C_s_-H → R-C_s_-OH → R-C_s_=O → R-C_s_OOH → R-H + 2H_2_O + CO_2_  (S11)

More broadly, since carbon materials often contain surface oxides to some extent, initial oxidation may begin from phenols, carbonyls, carboxylic acids, ethers, quinones, and lactones. However, H_2_O is still necessary to provide the source of oxygen for further oxidation (generating CO or CO_2_ gas). These surface groups can provide significant proton-involving pseudocapacitance, [11-17] as in the case of M-YP-50 (**Fig. S13**). On the other hand, uncontrolled reactions leading to further oxidation/corrosion reactions of the carbon material generating CO/CO_2_ may result in shedding of catalyst supported on carbon[9] or capacitance fading when carbon is the active material itself as in some aqueous supercapacitors.[11, 18] Therefore, oxidation/corrosion of carbon is commonly recognized as a degradation mechanism for electrochemical devices during cycling or sustained loading.

**Electrochemical Data.** Electrochemical properties were calculated from the cyclic voltammetry and galvanostatic charge–discharge curves in the same way as in Reference. [19]

Specifically, for a nonlinear Faradaic capacitor, the capacitance is obtained from the integrated form


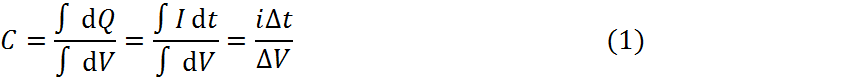


In the above, *i* is the average current during the CV/CC charging or discharging cycle, *ΔV* is the potential window, and *Δt* is the charging or discharging time.


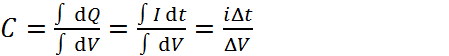


(*a*) CV test

Since d*V/*d*t=v* is a set constant (i.e., the scan rate), integration over the entire loop gives the average capacitance


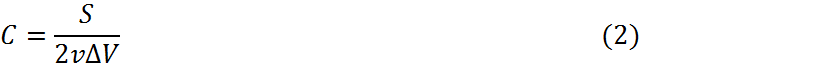


Here is the loop area. The specific capacitance is the capacitance divided by the mass of the electrode.


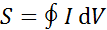

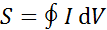

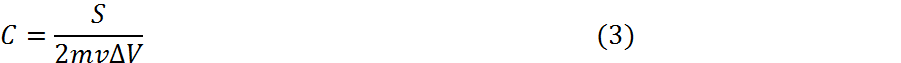


The surface capacitance is likewise obtained by dividing capacitance by the area of the electrode. And the specific capacity (mA h g^-1^) can be calculated by *S/2mv*

The energy density is obtained from the integral. To average over the charging/discharging cycle, absolute values are used in integration


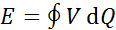

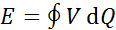

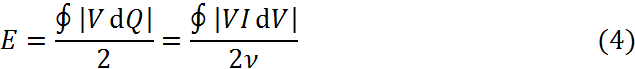


The specific energy is the energy divided by the mass of the electrode


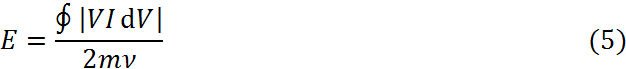


Similarly, the energy density is obtained by dividing the energy by the volume of the electrode.

(*b*) CC test

Since I is a set constant (i.e., the charging/discharging rate), i=I so Eq (1) reduces to


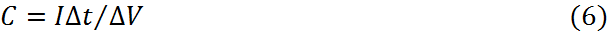


(For our Faradaic capacitance, the charging/discharging curves are rather linear, so ΔV/Δt is essentially the slope.) The specific capacitance is the capacitance divided by the mass of the electrode


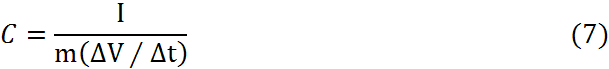


The specific surface capacitance is likewise obtained by dividing capacitance by the area of the electrode. And the specific capacity (mA h g^-1^) can be calculated by *IΔt/m*

The energy density is obtained from the integration


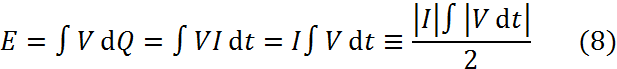


In the above, the last integral over both half cycles obtains an average.

The device energy and power are calculated considering only the weight of active materials Power (*P_wt_*) is obtained from *P_wt_*=*V*^2^/4*R*_ESR_, where V is the operating voltage and *R*_ESR_ is the equivalent serial resistance (ESR) of the device. ESR is obtained from the CC test by dividing the voltage drop (*V*_drop_) upon current reversal by twice the value of *I*, i.e., *R*_ESR_=*V*_drop_/2*I*.

**Table S4 Comparison of** **on some related carbon-based aqueous and non-aqueous supercapacitors.**

| Active Materials | Electrolyte | Energy density (Wh/kg) | Power density (kW/kg) | Capacitance  retention | Ref |
| --- | --- | --- | --- | --- | --- |
| OMFLC-N  M-YP-50 | 2M Li_2_SO_4_ | 127  28 | 237  34 | [93.8%@100k](mailto:93.8%25@100k)  84.5%@60k | This work |
| OMFLC-N  YP-50 | 0.5 M Li_2_SO_4_ | 63  12.5 | 44  25.5 | 80%@50k | [19] |
| NCNF | 1M KOH | 31.04 | 0.25 | 97%@10k | [20] |
| AC | EMIMBF_4_ | 88 | / | 96%@5k | [21] |
| N-doped SGC | 1M Na_2_SO_4_ | 25.7 | 35.6 | 97.2%@5k | [22] |
| MHCN | 1 M TEABF_4_ | 22.4 | ~60 | / | [23] |
| EM-CCG | EMIMBF_4_ | 47.9 | ~93 | 95%@300 h | [24] |
| Compressed-25 | EMIMBF_4_ | 63 | 12.75 | / | [25] |
| a-MEGO | BMIM BF_4_ | 70 | 75 | 97%@10k | [26] |
| FGN-300 | 1 M Na_2_SO_4_ | 26.4 | 16.6 | 134%@10k | [27] |
| Holey graphene | 6M KOH  EMIMBF_4_ | 35  127 | 7.5 | 95%@20k | [28] |
| C-0.75-900 | EMIBF_4_ | 92 | 200 | 92.2%@10k | [29] |
| 2D-HPCs | EMIMBF_4_ | 139 | 9.9 | 96%@10k | [30] |
| CS-HPGC | EMIM TFSI  KOH | 63.3  10.2 | 75  25 | 94.3%@10k  94%@10k | [31] |
| PGCs-13 | 1 M LiPF_6_ | 83.7 | 6.527 | 97.3%@10k | [32] |


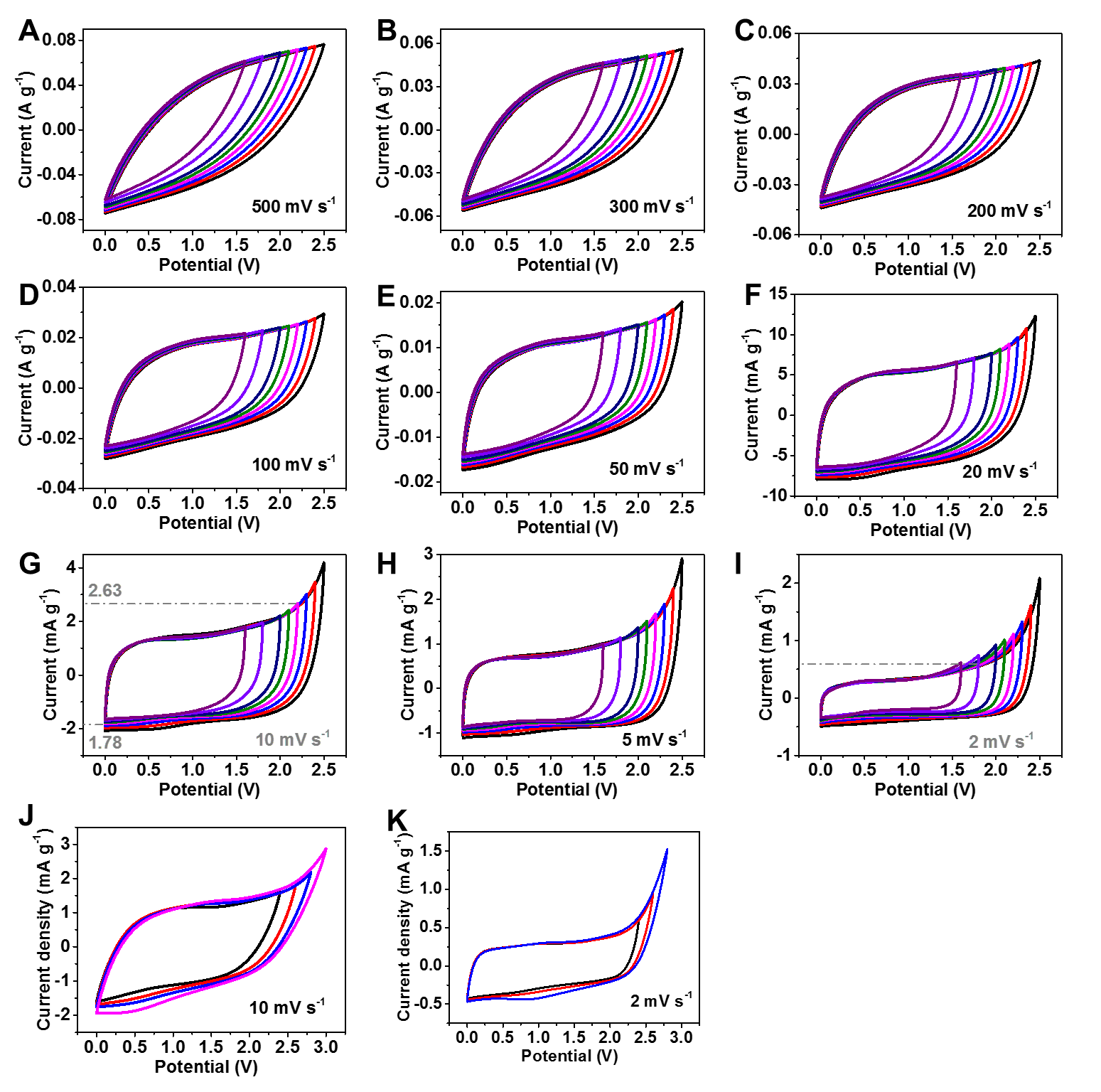


**Figure S1.** Symmetric cells with uncoated YP-50-CCF-GP electrodes in aqueous 2M Li_2_SO_4_ (pH~6.0). CV curves with different voltage (from 1.6 V to 2.4 V) windows at the scan rate of (**A**) 500 mV s^-1^, (**B**) 300 mV s^-1^, (**C**) 200 mV s^-1^, (**D**) 100 mV s^-1^, (**E**) 50 mV s^-1^, (**F**) 20 mV s^-1^, (**G**) 10 mV s^-1^, (**H**) 5 mV s^-1^, (**I**) 2 mV s^-1^..

**Figure S2. Periodic graphene** models used in our calculations. (**A**) Pristine, (**B**) N-doped, and (**C**) O-doped graphene. C, N, O, and H are represented in brown, grey, red and white, respectively. The unit cells are also framed out along a and b axis.


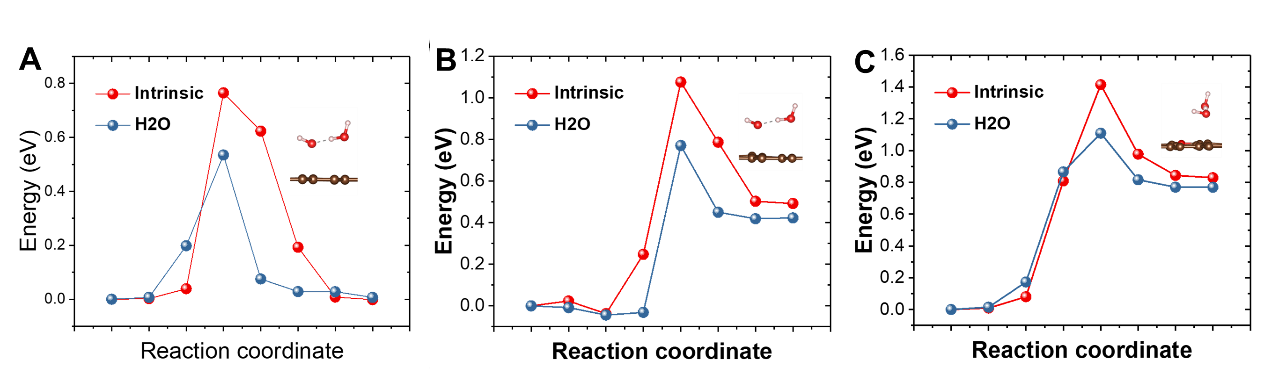


**Figure S3.** Calculated energy barrier for OH diffusion by NEB method and corresponding transition state mediated by water for (A) pristine, (B,) N-doped, and (C) O-doped graphene. C, N, O, and H are represented in brown, grey, red and white, respectively.


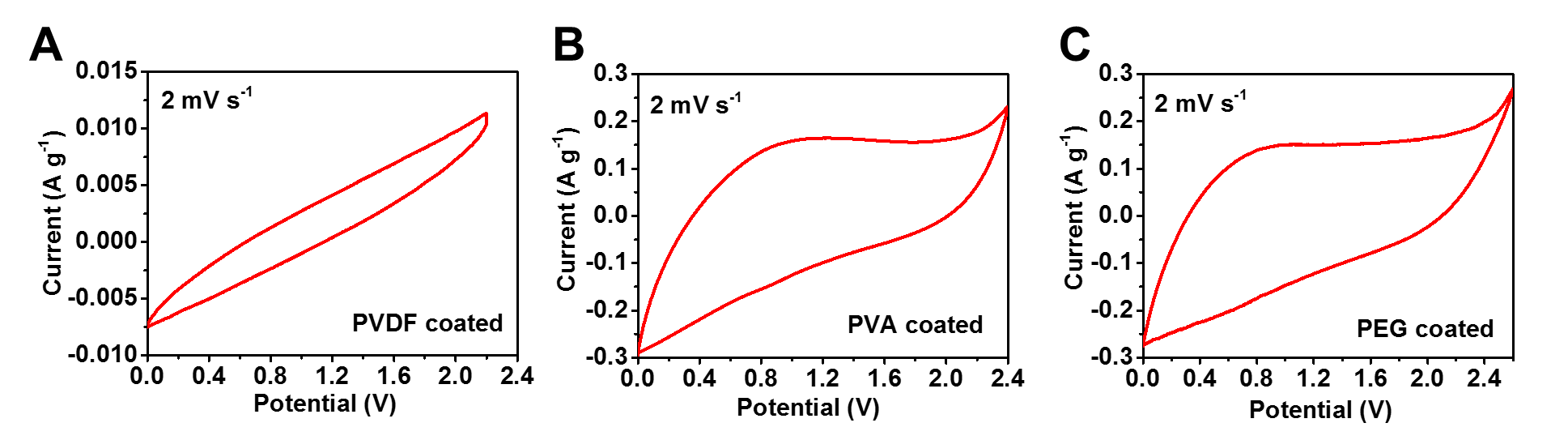


**Figure S4.** Cyclic voltammetry curves of symmetric devices, which contain 2 mg YP-50 active carbon on each electrode, then dip-coated with (**A**) PVDF, (**B**) PVA, and (**C**) PEG. See **Methods**. Electrolyte used was 2M Li_2_SO_4_ (pH~6) aqueous solution.


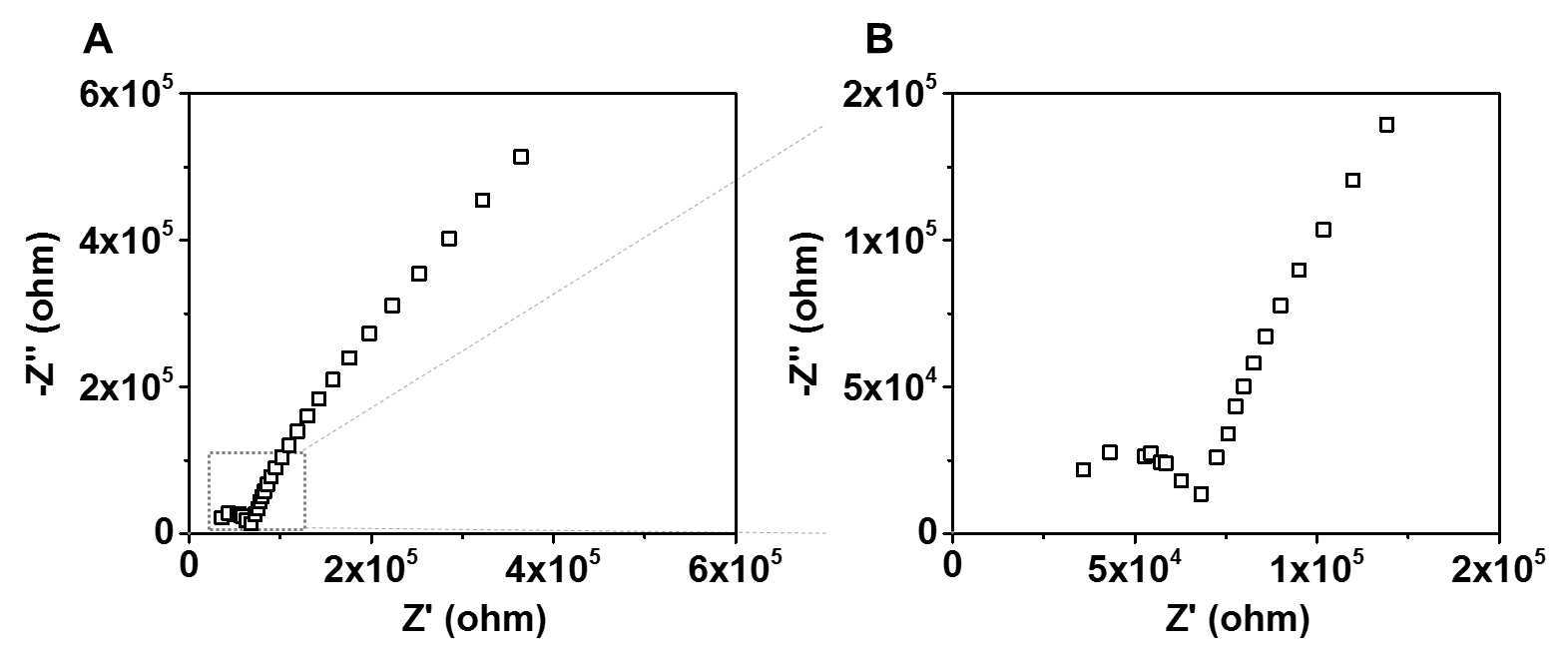


**Figure S5.** (**A**) Nyquist plot of complex impedance from 0.01 Hz to 100 kHz due to proton conduction of dry PAA film, between stainless steel electrodes, and (**B**) its enlarged view.


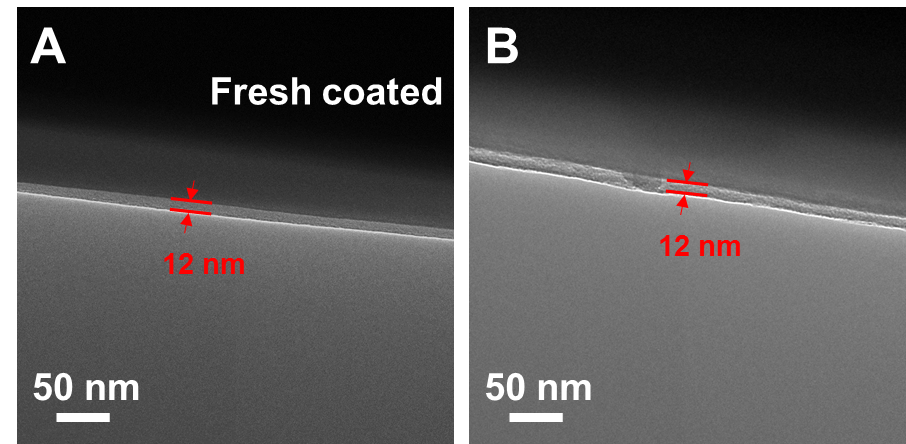


**Figure S6.** TEM images of (**A**) freshly coated CCF electrode (15 coating cycles) and (**B**) (A) after 2,000 cycles from −1.2 V to 1.0 V at 50 mV s^-1^. PAA coating thickness is ~15 nm in both.


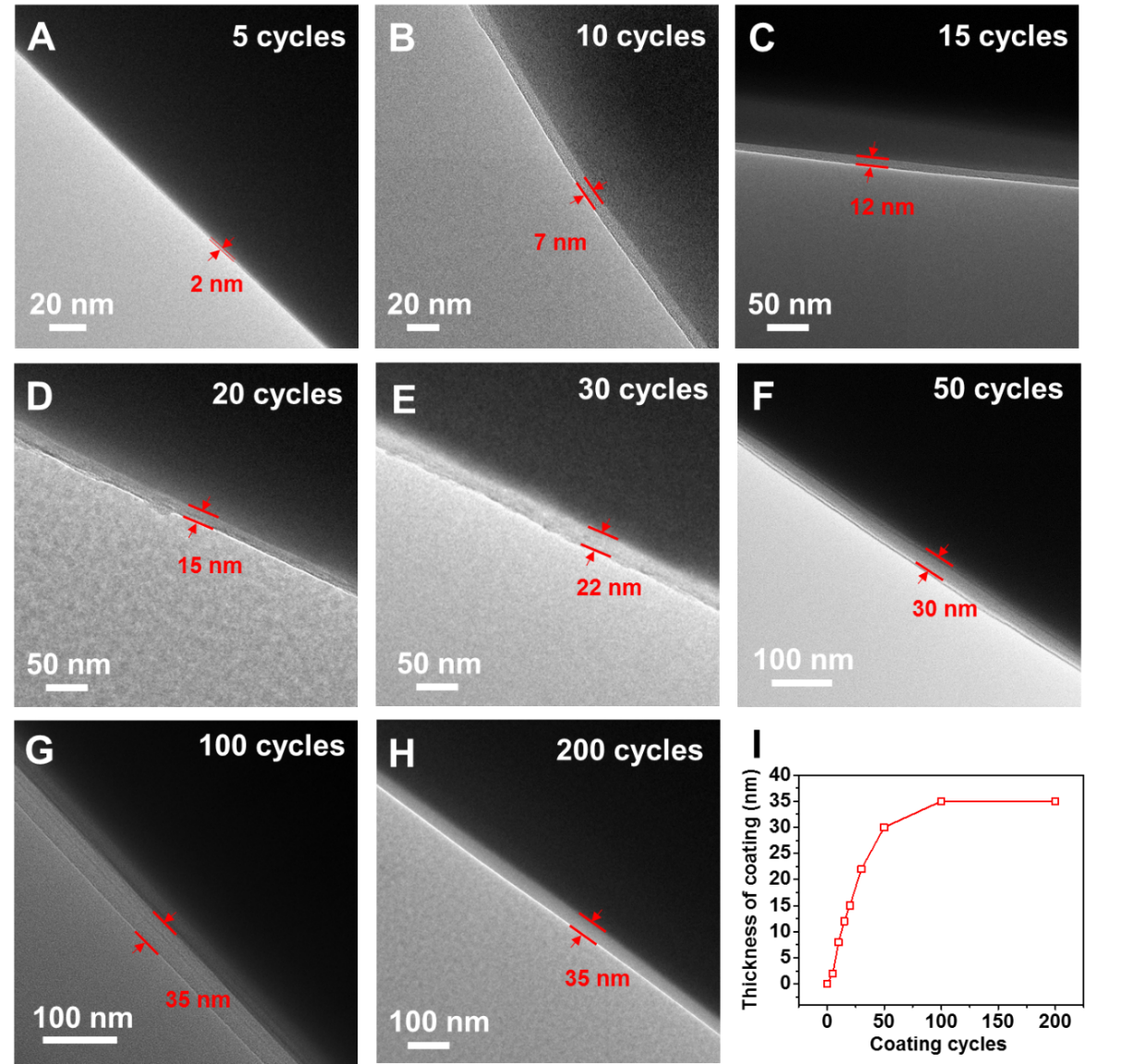


**Figure S7.** TEM images of coated CCF electrodes with (A) 5, (B) 10, (C) 15, (D) 20, (E) 30, (F) 50, (G) 100, and (H) 200 coating cycles. (I) PAA thickness versus number of cycles.


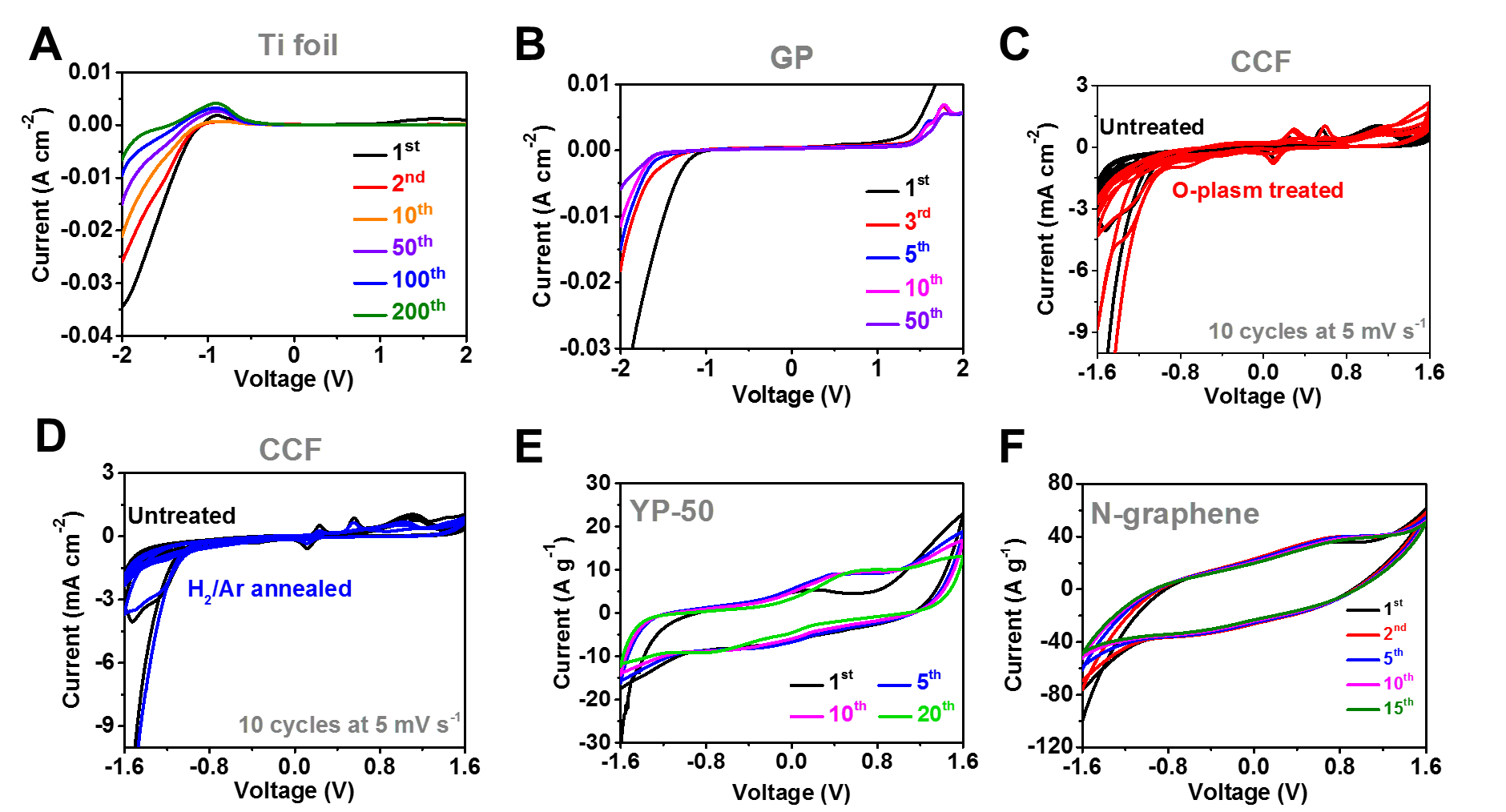


**Figure S8.** Cyclic voltammetry (CV) curve during PAA coating showing electrochemical window (ECW) evolution of **(A)** Ti foil and (**B**) graphite paper (GP), in 2M Mg:2AA solution using cyclic voltammetry (CV) cycles between −2.0 and 2.0 V at scan rate of 50 mV s^-1^. Similarly, CV cycles between –1.6 and 1.6 V of (**C**) unoxidized vs. oxygen plasma pre-oxidized CCF and (**D**) unoxidized vs. H_2_/Ar pre-reduced CCF, in 2M Mg:2AA solution at scan rate of 5 mV s^-1^. Similarly, CV cycles between –1.6 and 1.6 V of (**E**) YP-50 and (**F**) N-graphene loaded CCF-GP electrodes, in 2M Mg:2AA solution at scan rate of 50 mV s^-1^.


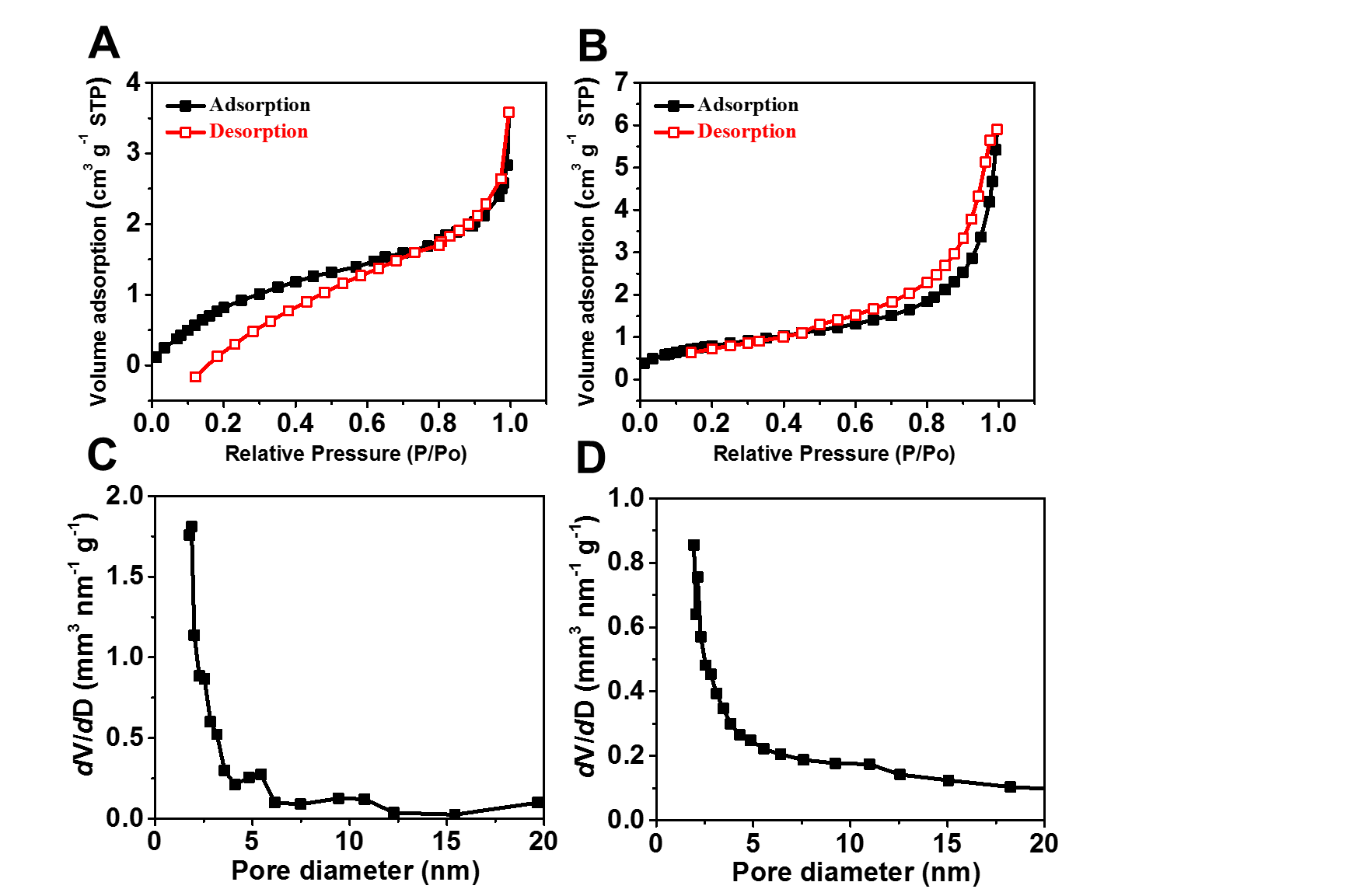
**Figure S9.** Nitrogen adsorption-desorption isotherms of (A) uncoated and (B) PAA coated (50 cycles) CCF electrodes. Pore size distribution of (C) uncoated and (D) PAA coated (50 cycles).


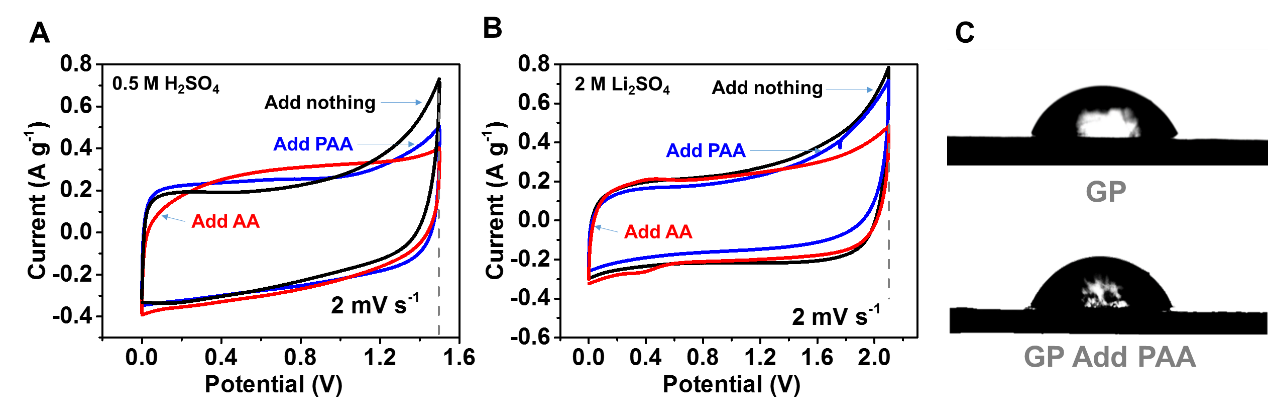


**Figure S10.** CV curves (scan rate of 2 mV s^-1^) of assembled symmetric cells, which use YP-50 as active material on CG as current collector, and (**A**) 0.5 M H_2_SO_4_ or (**B**) 2 M Li_2_SO_4_ as electrolyte. Nothing, AA or PAA (polymerized in-house) at 40 g L^-1^ was added into the electrolyte. Since AA and PAA are weak acids, the pH of 0.5 M H_2_SO_4_ is barely affected. pH~6 obtained for 2 M Li_2_SO_4_ by adding 2 M LiOH aqueous solution. (C) Water droplet on the uncoated graphite paper (GP) and the GP electrode cycled in the PAA added electrolyte.


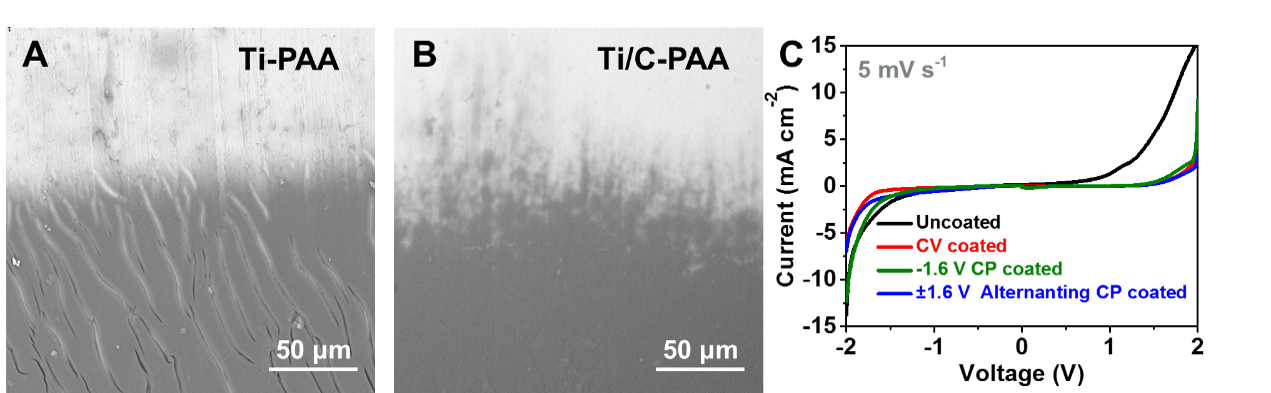


**Figure S11.** PAA on different electrodes exhibits different adhesion. (**A**) Hairline cracks appear on lower half of untreated Ti electrode. (**B**) no crack on carbon-pre-coated Ti electrode. (**C**) Linear sweep voltammetry (LSV) curves in 2M Li_2_SO_4_ (pH~6) aqueous electrolyte of uncoated (black) and coated CCF electrodes. Three coating modes were used: CV-coated (−1.6 V to 1.6 V, 50 mV s^-1^, 50 cycles), constant-potential-coated (CP, −1.6 V, 3,200 s), and alternating-CP-coated (−1.6 V, 10 s; 1.6 V, 10 s; 320 cycles). CV-coated electrode has widest ECW.


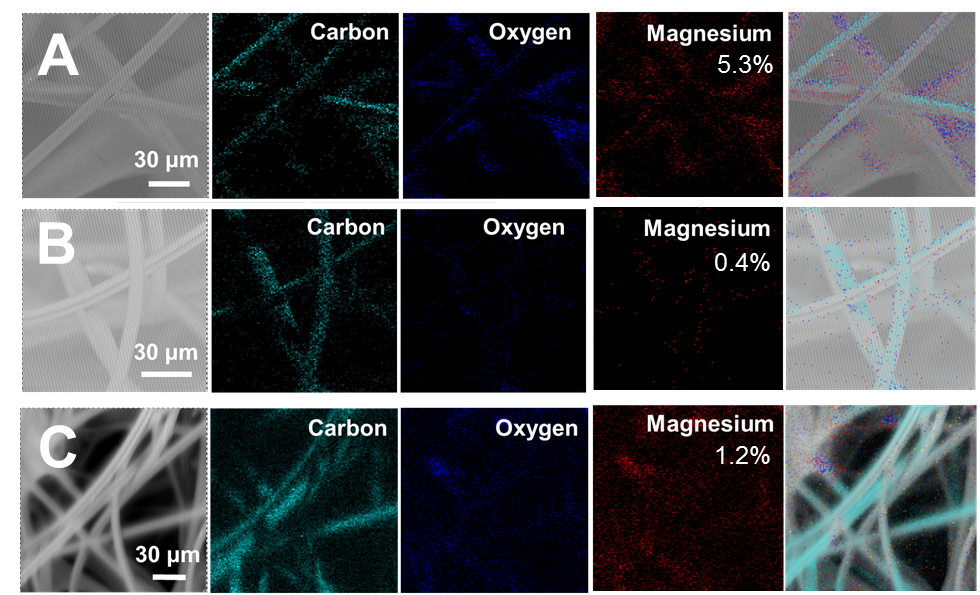


**Figure S12.** SEM micrographs and elemental mapping of PAA coating on CCF electrodes. Initial coating performed by 50 cyclic voltammetry cycles between −2.0 V to 2.0 V at 50 mV s^-1^ in 2M Mg: 2AA electrolyte. (**A**) As-coated CCF (upper left) has uniform distribution of C, O, and Mg, last micrograph showing their combination. (**B**) Same as (**A**) but after another 50 cycles between −1.0 V to 1.0 V in an Mg-free 0.5 M H_2_SO_4_. (**C**) Same as (**B**) but after further 50 cycles between −1.0 V to 1.0 V, this time in a 2 M MgSO_4_ electrolyte.


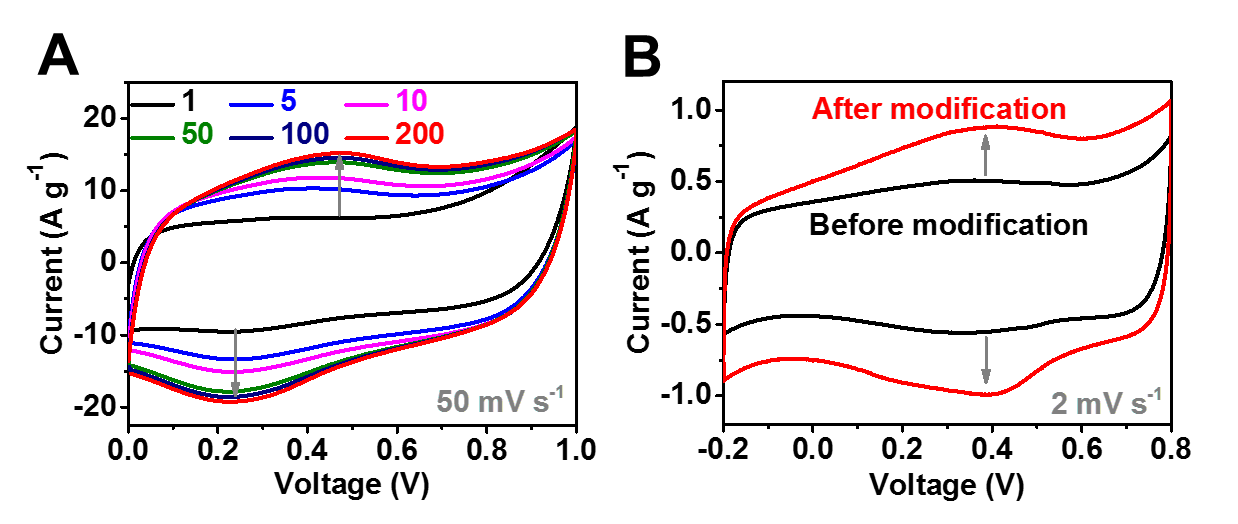


**Figure S13.** (**A**) Successive cyclic voltammetry curves of YP-50 loaded CCF-GP electrodes cycled 200 times in 1M H_2_SO_4_ aqueous electrolyte at 50 mV s^-1^ resulting in oxidizing YP-50 to become modified YP-50 (M-YP-50 for short) with prominent redox peak at 0.4 V. (**B**) Cyclic voltammetry curves of M-YP-50 vs. unmodified M-YP-50.


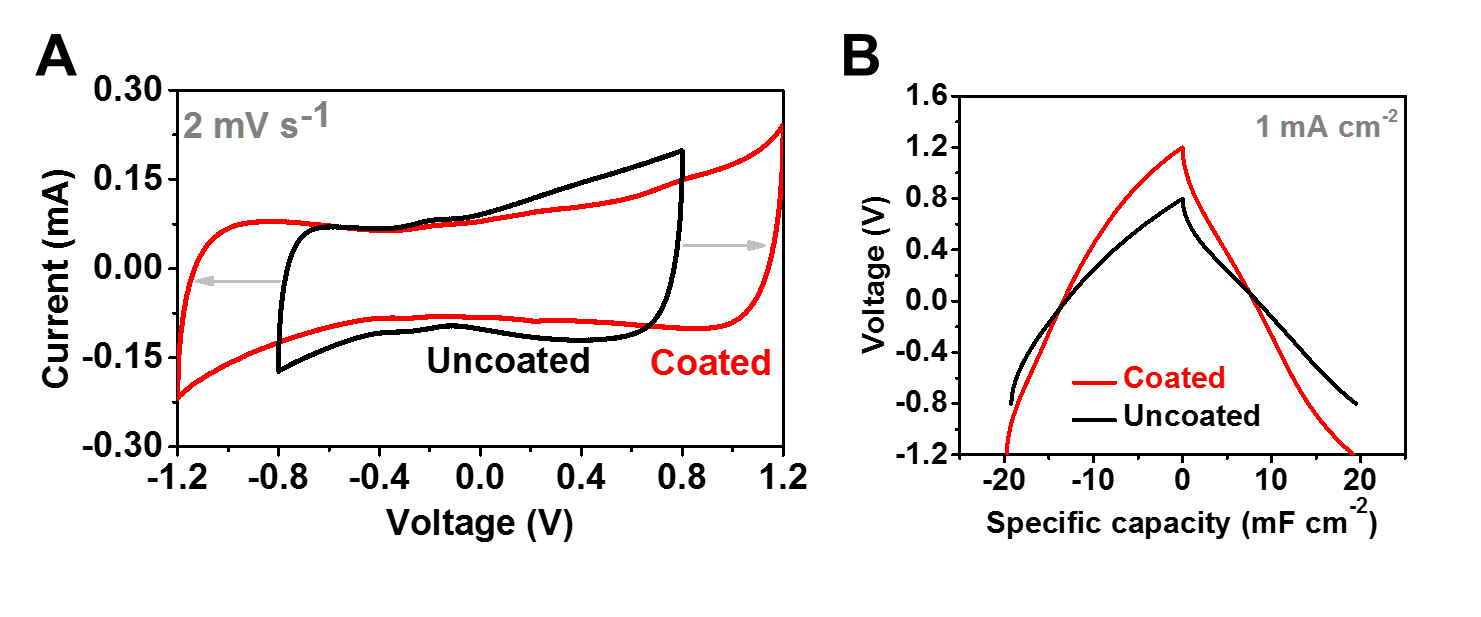


**Figure S14.** (**A**) Cyclic voltammetry of uncoated and PAA-coated graphite paper. (**B**) Corresponding galvanic charge/discharge curves.


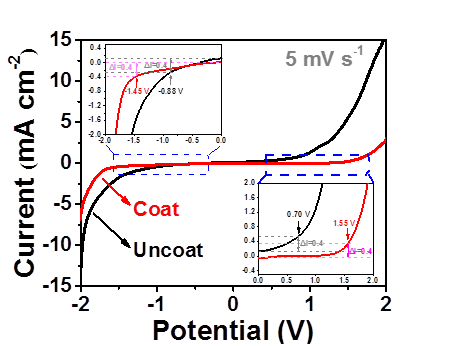


**Figure S15.** Linear sweep voltammetry curves of uncoated and PAA-coated CCF electrode tested in 2M Li_2_SO_4_ (pH~6) electrolyte at scan rate of 5 mV s^-1^. **Insets**: enlarged views of regions near anodic and cathodic extremes. Coating performed in 2M Mg: 2AA solution by cycling between −1.6 to 1.6 V at scan rate of 50 mV s^-1^.


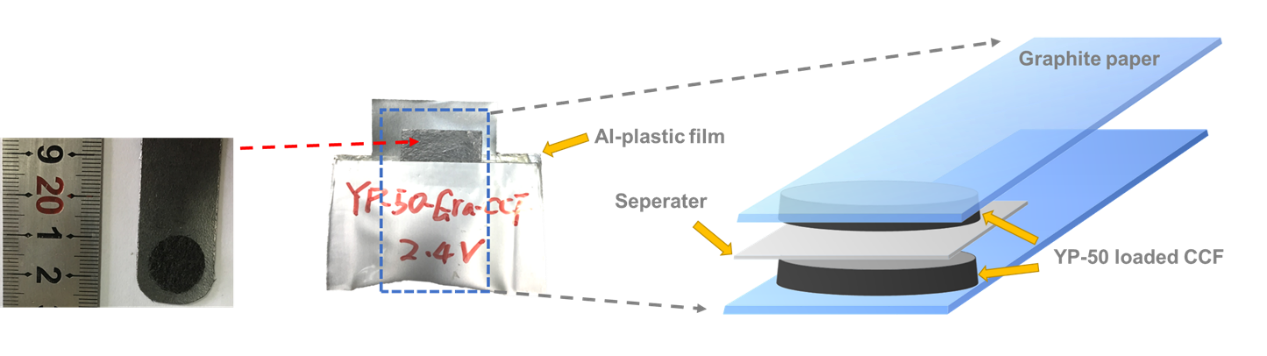


**Figure S16.** Assembled soft packaged symmetric cell, wit two symmetric PAA-coated YP-50-CCF-GP electrodes.


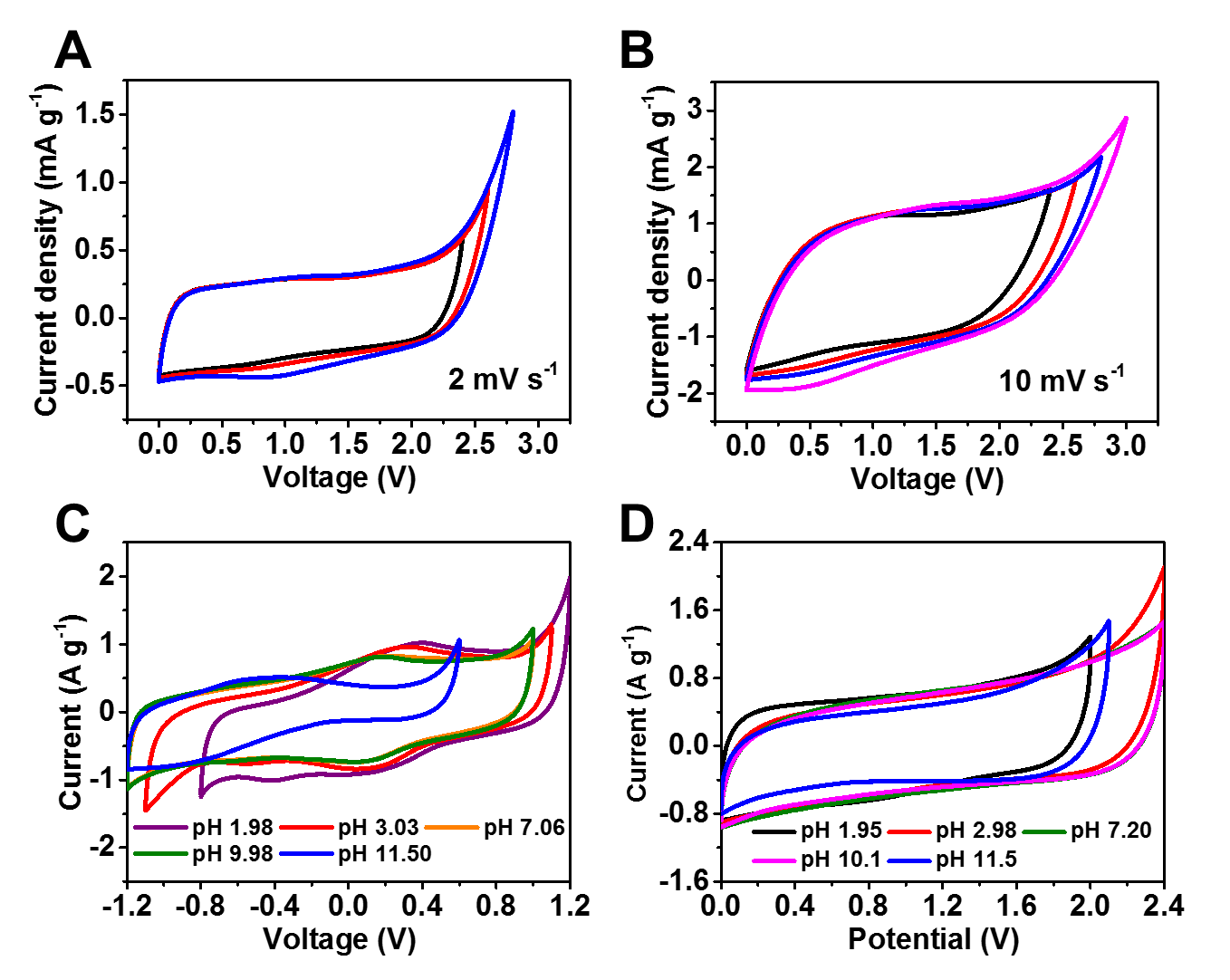


**Figure S17.** CV curves of the cell with PAA coated YP-50-CCF-GP electrodes at scan rate of (**A**) 2mV s^-1^ and (**B**) 10 mV s^-1^. Cyclic voltammetry curves vs. pH of PAA-coated N-graphene-CCF-GP at 2 mV s^-1^ in (**C**) three-electrode and (**D**) symmetrical cell configuration. 2M Li_2_SO_4_ electrolyte adjusted to different pH.


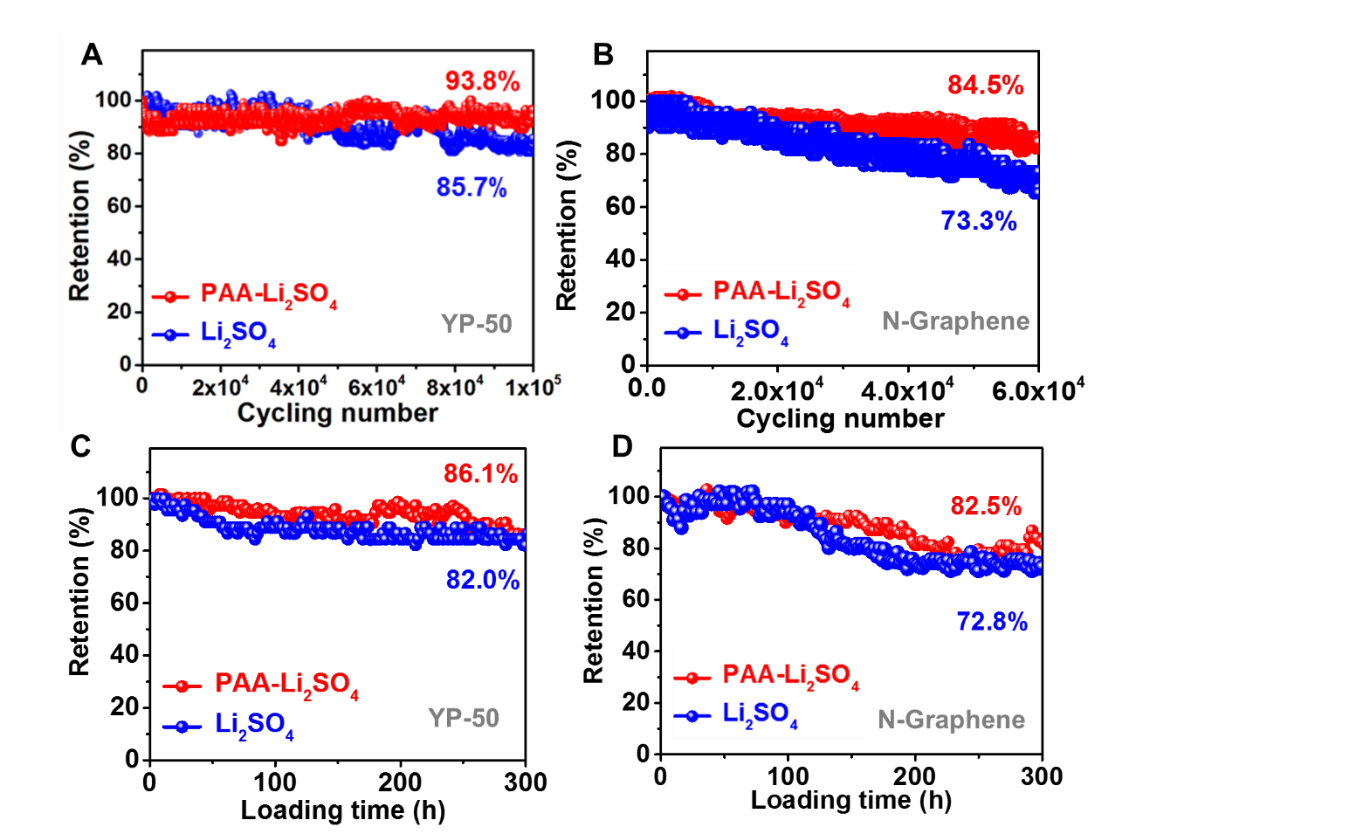


**Figure S18.** (**A-B**) Cycling durability of YP-50/N-graphene-loaded symmetric soft package cell during galvanostatic charging–discharging at 1 A g^-1^ for 10^5^ cycles. Coated electrode data labeled as PAA-Li_2_SO_4_ cycled under 2.4 V, uncoated ones as Li_2_SO_4_ cycled under 1.8 V. (**C-D**) Same as (**A-B**) but under sustained loading of 2.4 V forcoated cells and 1.8 V for uncoated cells for 300 h. Electrolyte: neutral aqueous solution of 2 M Li_2_SO_4_.


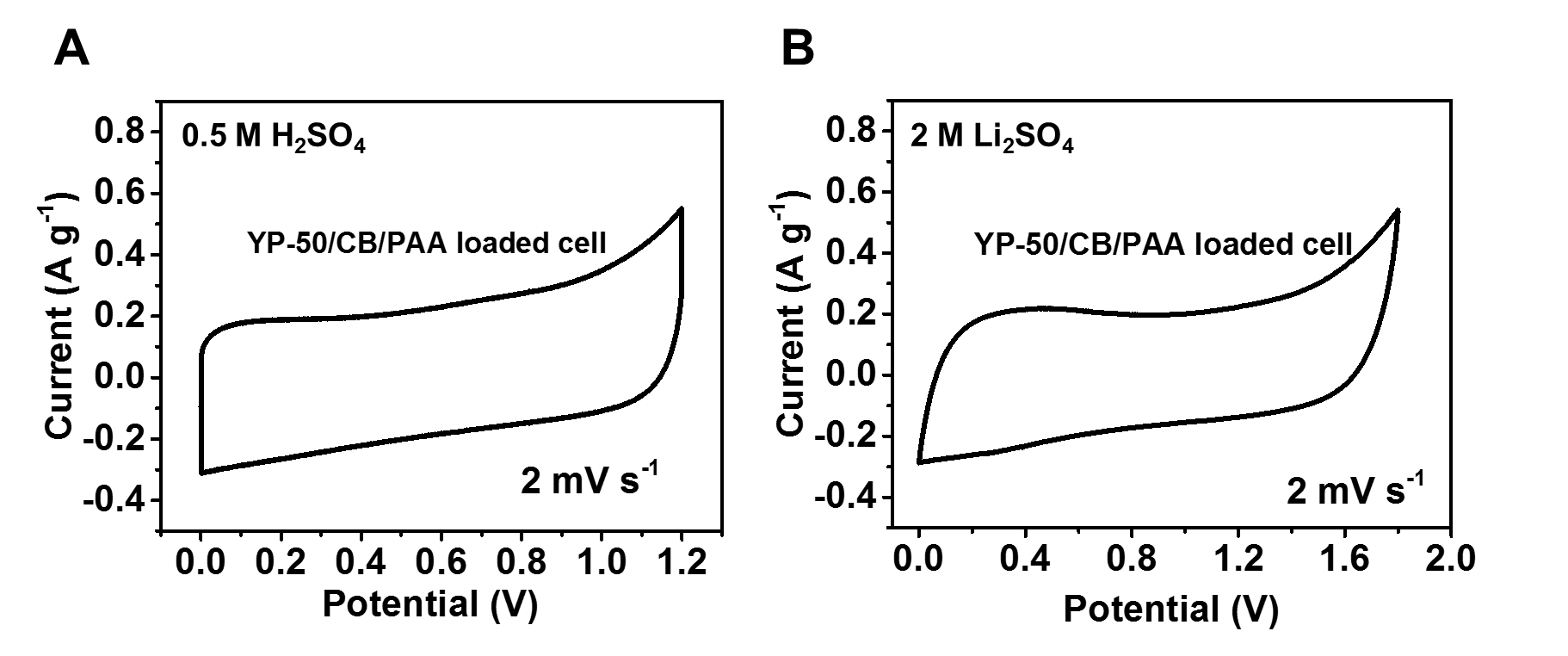


**Figure S19.** Cyclic voltammetry curves (scan rate of 2 mV s^-1^) of assembled cells, which use YP-50 as active material, carbon black (CB) as conductive additive, Ti foil as current collector, PAA as binder, and (**A**) 0.5 M H_2_SO_4_ or (**B**) 2 M Li_2_SO_4_ as electrolyte.


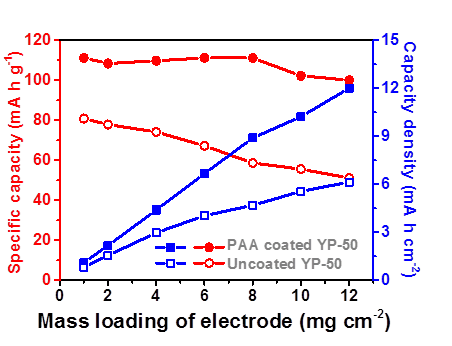


**Figure S20.** Linear specific gravimetric (left) and surface (right) capacitance (at 1 A g^−1^) of symmetric electrochemical cell devices (counting active materials weight only) versus areal mass loading of YP-50 in 2M Li_2_SO_4_ electrolyte.

**
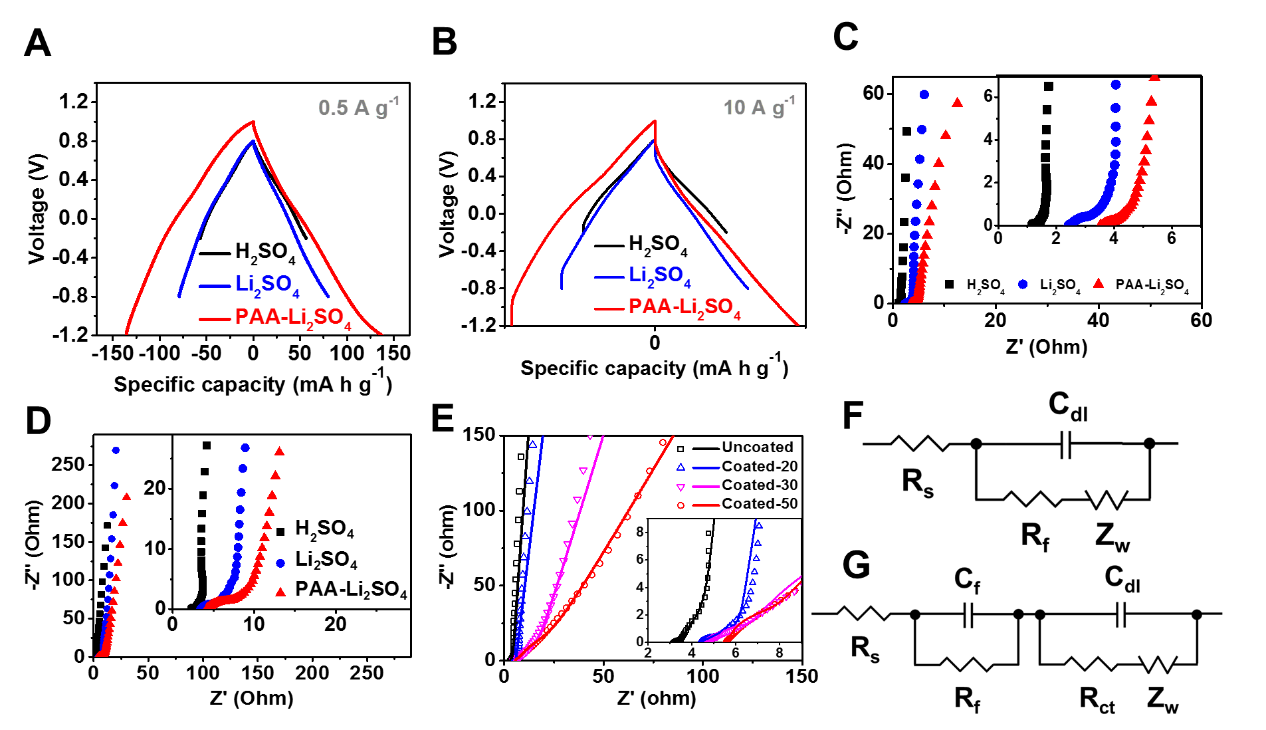
**

**Figure S21.** Galvanostatic charge-discharge curves at charging/discharging rates of (**A**) 0.5 A g^-1^ and (**B**) and 10 A g^-1^, showing larger voltage drop upon reversal of current direction in coated electrode. (**C-D**) Nyquist plots of complex impedance from 0.01 Hz to 10^5^ Hz in (**C**) three-electrode and (**D**) symmetric cell configuration; **Inset**: enlarged view of high frequency region. Data shown are from uncoated YP-50-CCF-GP electrode tested in 0.5 M H_2_SO_4_ electrolyte (labeled as “H_2_SO_4_”) and pH~6 2M Li_2_SO_4_ electrolyte (labeled as “Li_2_SO_4_”), and coated YP-50-CCF-GP electrodes tested in pH~6 2M Li_2_SO_4_ electrolyte (labeled as “Li_2_SO_4_-PAA”. (**E**) Nyquist plots from 0.01 Hz to 10^5^ Hz of uncoated and coated YP-50-CCF-GP electrodes in three-electrode configuration in pH~6 2M Li_2_SO_4_ electrolyte. Solid curves from simulation using models (**F-G**) below. **Inset**: magnified view of high frequency regions. Coating in (E) obtained by 20-50 voltammmetry cycles from −1.6 V to 1.6 V at 50 mV s^-1^. Equivalent circuits for (**F**) capacitor-like and (**G**) battery-like behavior. In (E), simulation uses model (F) for uncoated and coated-20, and (G) for coated-30 and coated-50.


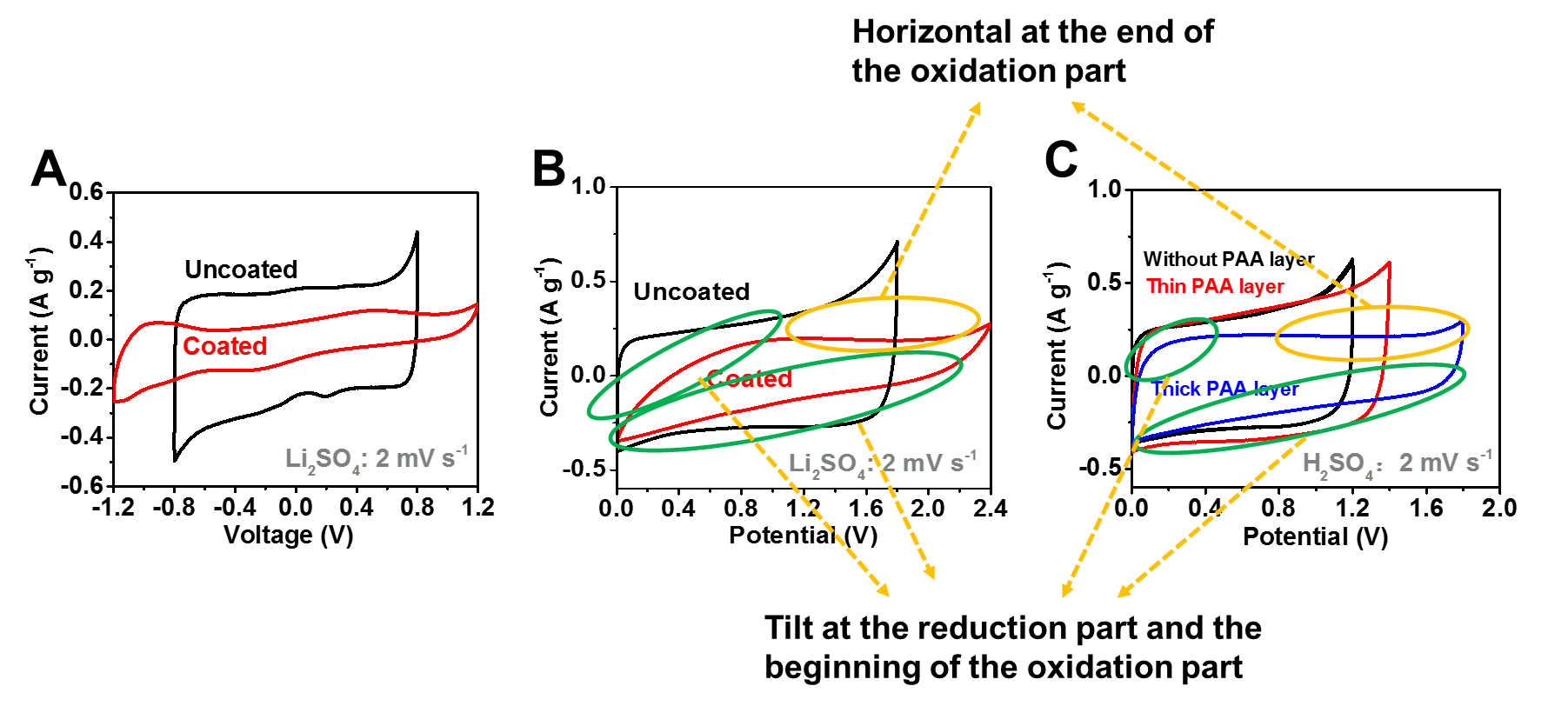


**Figure S22.** Cyclic voltammetry, at 2 mV s^-1^, of uncoated and variously PAA-coated YP-50 CCF-GP electrodes tested in 2M Li_2_SO_4_ (pH~6) in (**A**) three-electrode and **(B)** symmetrical cell configuration, and (**C**) in 0.5M H_2_SO_4_ in symmetrical cell configuration. PAA coating obtained by CV cycling from −1.6 V to 1.6 V at 50 mV s^-1^, for (**A**) 50 cycles, (**B**) 40 cycles, and (**C**) 10 cycles for thin coat and 30 cycles for thick coat. Severe shear of CV loops is expected if the decreased capacitance results from voltage sharing of a serial resistance. However, as shown in these data, the loops remain flat at the end of the oxidation part (yellow circles) regardless of thickness of PAA coating.


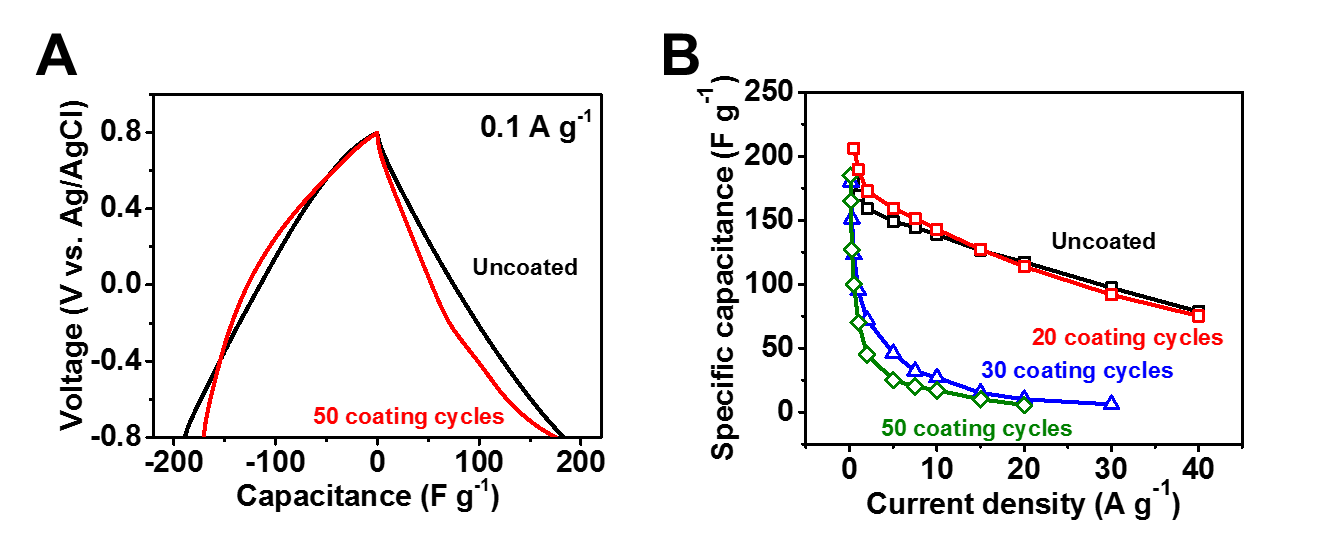


**Figure S23.** Rate capability from galvanostatic test results of uncoated, 20-cycle coated, 30-cycle coated, and 50-cycle coated YP-50-CCF-GP electrodes. Tested at −0.8 V to 0.8 V in neutral 2 M Li_2_SO_4_.

**References:**

[1] Y. Jiao; Y. Zheng; K. Davey; S. Z. Qiao, "Activity origin and catalyst design principles for electrocatalytic hydrogen evolution on heteroatom-doped graphene,"*Nature Energy*, vol. *1*, no., pp., 2016.

[2] A. Kozbial; F. Zhou; Z. T. Li; H. T. Liu; L. Li, "Are Graphitic Surfaces Hydrophobic?,"*Accounts of Chemical Research*, vol. *49*, no. 12, pp. 2765-2773, 2016.

[3] M. T. Li; L. P. Zhang; Q. Xu; J. B. Niu; Z. H. Xia, "N-doped graphene as catalysts for oxygen reduction and oxygen evolution reactions: Theoretical considerations,"*Journal of Catalysis*, vol. *314*, no., pp. 66-72, 2014.

[4] J. K. Norskov; T. Bligaard; A. Logadottir; J. R. Kitchin; J. G. Chen; S. Pandelov; J. K. Norskov, "Trends in the exchange current for hydrogen evolution,"*Journal of The Electrochemical Society*, vol. *152*, no. 3, pp. J23-J26, 2005.

[5] Z. W. Seh; J. Kibsgaard; C. F. Dickens; I. B. Chorkendorff; J. K. Norskov; T. F. Jaramillo, "Combining theory and experiment in electrocatalysis: Insights into materials design,"*Science*, vol. *355*, no. 6321, pp., 2017.

[6] G. Henkelman; B. P. Uberuaga; H. Jonsson, "A climbing image nudged elastic band method for finding saddle points and minimum energy paths,"*Journal of Chemical Physics*, vol. *113*, no. 22, pp. 9901-9904, 2000.

[7] vol., no., pp., !!! INVALID CITATION !!! [7].

[8] L. R. Merte; G. W. Peng; R. Bechstein; F. Rieboldt; C. A. Farberow; L. C. Grabow; W. Kudernatsch; S. Wendt; E. Laegsgaard; M. Mavrikakis; F. Besenbacher, "Water-Mediated Proton Hopping on an Iron Oxide Surface,"*Science*, vol. *336*, no. 6083, pp. 889-893, 2012.

[9] K. H. Kangasniemi; D. A. Condit; T. D. Jarvi, "Characterization of vulcan electrochemically oxidized under simulated PEM fuel cell conditions,"*Journal of The Electrochemical Society*, vol. *151*, no. 4, pp. E125-E132, 2004.

[10] Q. Gao; L. Demarconnay; E. Raymundo-Pinero; F. Beguin, "Exploring the large voltage range of carbon/carbon supercapacitors in aqueous lithium sulfate electrolyte,"*Energy & Environmental Science*, vol. *5*, no. 11, pp. 9611-9617, 2012.

[11] K. Kinoshita; J. A. S. Bett, "Potentiodynamic Analysis of Surface Oxides on Carbon-Blacks,"*Carbon*, vol. *11*, no. 4, pp. 403-411, 1973.

[12] K. Kinoshita, *Carbon: electrochemical and physicochemical properties*. John Wiley Sons,New York, NY; None: 1988; p Medium: X; Size: Pages: 541.

[13] T. Momma; X. J. Liu; T. Osaka; Y. Ushio; Y. Sawada, "Electrochemical modification of active carbon fiber electrode and its application to double-layer capacitor,"*Journal of Power Sources*, vol. *60*, no. 2, pp. 249-253, 1996.

[14] M. G. Sullivan; B. Schnyder; M. Bartsch; D. Alliata; C. Barbero; R. Imhof; R. Kotz, "Electrochemically modified glassy carbon for capacitor electrodes characterization of thick anodic layers by cyclic voltammetry, differential electrochemical mass spectrometry, spectroscopic ellipsometry, X-ray photoelectron spectroscopy, FTIR, and AFM,"*Journal of The Electrochemical Society*, vol. *147*, no. 7, pp. 2636-2643, 2000.

[15] E. Frackowiak; F. Beguin, "Carbon materials for the electrochemical storage of energy in capacitors,"*Carbon*, vol. *39*, no. 6, pp. 937-950, 2001.

[16] H. B. Xu; X. Z. Fan; Y. H. Lu; L. A. Zhong; X. F. Kong; J. Wang, "Preparation of an electrochemically modified graphite electrode and its electrochemical performance for pseudo-capacitors in a sulfuric acid electrolyte,"*Carbon*, vol. *48*, no. 11, pp. 3300-3303, 2010.

[17] X. Z. Fan; Y. H. Lu; H. B. Xu; X. F. Kong; J. Wang, "Reversible redox reaction on the oxygen-containing functional groups of an electrochemically modified graphite electrode for the pseudo-capacitance,"*Journal of Materials Chemistry*, vol. *21*, no. 46, pp. 18753-18760, 2011.

[18] M. L. He; K. Fic; E. Frackowiak; P. Novak; E. J. Berg, "Ageing phenomena in high-voltage aqueous supercapacitors investigated by in situ gas analysis,"*Energy & Environmental Science*, vol. *9*, no. 2, pp. 623-633, 2016.

[19] T. Q. Lin; I. W. Chen; F. X. Liu; C. Y. Yang; H. Bi; F. F. Xu; F. Q. Huang, "Nitrogen-doped mesoporous carbon of extraordinary capacitance for electrochemical energy storage,"*Science*, vol. *350*, no. 6267, pp. 1508-1513, 2015.

[20] H. Chen; T. Liu; J. Mou; W. Zhang; Z. Jiang; J. Liu; J. Huang; M. Liu, "Free-standing N-self-doped carbon nanofiber aerogels for high-performance all-solid-state supercapacitors,"*Nano Energy*, vol. *63*, no., pp. 103836, 2019.

[21] L. Zhang; F. Zhang; X. Yang; K. Leng; Y. Huang; Y. Chen, "High-Performance Supercapacitor Electrode Materials Prepared from Various Pollens,"*Small*, vol. *9*, no. 8, pp. 1342-1347, 2013.

[22] J. Yan; Q. Wang; C. Lin; T. Wei; Z. Fan, "Interconnected Frameworks with a Sandwiched Porous Carbon Layer/Graphene Hybrids for Supercapacitors with High Gravimetric and Volumetric Performances,"*Advanced Energy Materials*, vol. *4*, no. 13, pp. 1400500, 2014.

[23] G.-P. Hao; A.-H. Lu; W. Dong; Z.-Y. Jin; X.-Q. Zhang; J.-T. Zhang; W.-C. Li, "Sandwich-Type Microporous Carbon Nanosheets for Enhanced Supercapacitor Performance,"*Advanced Energy Materials*, vol. *3*, no. 11, pp. 1421-1427, 2013.

[24] X. Yang; C. Cheng; Y. Wang; L. Qiu; D. Li, "Liquid-Mediated Dense Integration of Graphene Materials for Compact Capacitive Energy Storage,"*Science*, vol. *341*, no. 6145, pp. 534-537, 2013.

[25] S. Murali; N. Quarles; L. L. Zhang; J. R. Potts; Z. Tan; Y. Lu; Y. Zhu; R. S. Ruoff, "Volumetric capacitance of compressed activated microwave-expanded graphite oxide (a-MEGO) electrodes,"*Nano Energy*, vol. *2*, no. 5, pp. 764-768, 2013.

[26] Y. W. Zhu; S. Murali; M. D. Stoller; K. J. Ganesh; W. W. Cai; P. J. Ferreira; A. Pirkle; R. M. Wallace; K. A. Cychosz; M. Thommes; D. Su; E. A. Stach; R. S. Ruoff, "Carbon-Based Supercapacitors Produced by Activation of Graphene,"*Science*, vol. *332*, no. 6037, pp. 1537-1541, 2011.

[27] J. Yan; Q. Wang; T. Wei; L. Jiang; M. Zhang; X. Jing; Z. Fan, "Template-Assisted Low Temperature Synthesis of Functionalized Graphene for Ultrahigh Volumetric Performance Supercapacitors,"*ACS nano*, vol. *8*, no. 5, pp. 4720-4729, 2014.

[28] Y. X. Xu; Z. Y. Lin; X. Zhong; X. Q. Huang; N. O. Weiss; Y. Huang; X. F. Duan, "Holey graphene frameworks for highly efficient capacitive energy storage,"*Nature Communications*, vol. *5*, no., pp., 2014.

[29] J. Li; N. Wang; J. Tian; W. Qian; W. Chu, "Cross-Coupled Macro-Mesoporous Carbon Network toward Record High Energy-Power Density Supercapacitor at 4 V,"*Advanced Functional Materials*, vol. *28*, no. 51, pp. 1806153, 2018.

[30] L. Yao; Q. Wu; P. X. Zhang; J. M. Zhang; D. R. Wang; Y. L. Li; X. Z. Ren; H. W. Mi; L. B. Deng; Z. J. Zheng, "Scalable 2D Hierarchical Porous Carbon Nanosheets for Flexible Supercapacitors with Ultrahigh Energy Density,"*Advanced Materials*, vol. *30*, no. 11, pp., 2018.

[31] Y. N. Gong; D. L. Li; Q. Fu; Y. P. Zhang; C. X. Pan, "Nitrogen Self-Doped Porous Carbon for High-Performance Supercapacitors,"*Acs Applied Energy Materials*, vol. *3*, no. 2, pp. 1585-1592, 2020.

[32] G. C. Li; Z. L. Yin; H. J. Guo; Z. X. Wang; G. C. Yan; Z. W. Yang; Y. Liu; X. B. Ji; J. X. Wang, "Metalorganic Quantum Dots and Their Graphene-Like Derivative Porous Graphitic Carbon for Advanced Lithium-Ion Hybrid Supercapacitor,"*Advanced Energy Materials*, vol. *9*, no. 2, pp., 2019.
